# Correction: Critical role of deadenylation in regulating poly(A) rhythms and circadian gene expression

**DOI:** 10.1371/journal.pcbi.1009065

**Published:** 2021-05-26

**Authors:** Xiangyu Yao, Shihoko Kojima, Jing Chen

In Fig 5 the image shown in part A is incorrect. The authors have provided a corrected version here.

**Fig 5:**
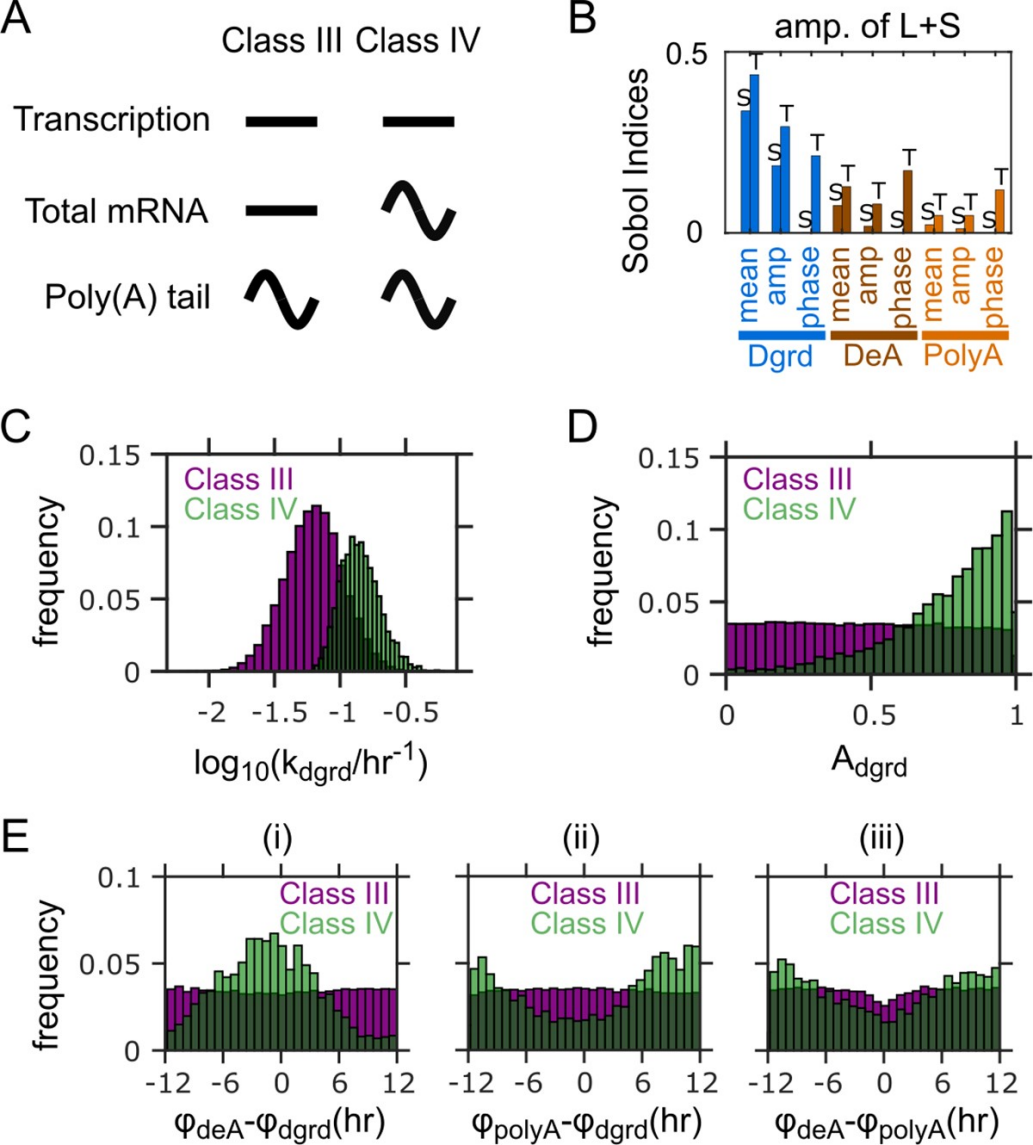
Factors distinguishing between Class III and Class IV PAR mRNAs. (A) Characteristics of Class III and the hypothetical Class IV mRNAs. (B) Sobol indices for the amplitude of L+S (i.e., total mRNA abundance) for the model without rhythmic transcription. Bars with “S” on top: single Sobol indices. Bars with “T” on top: total Sobol indices. (C) Distributions of mean mRNA degradation rates for the two classes. (D) Distributions of relative amplitudes of degradation for the two classes. (E) Distributions of peak phase differences (i) between deadenylation and degradation, (ii) between polyadenylation and degradation, and (iii) between deadenylation and polyadenylation for the two classes. Results in (C-E) from 100,000 simulations with parameters randomly sampled according to **Table 1**, but without rhythmic transcription (A_trsc_ = 0). Parameter sets with ≥0.2 relative amplitude in L/S ratio and <0.2 relative amplitude in L+S are defined as Class III, while those with and ≥0.2 relative amplitude in both L/S ratio and L+S are defined as Class IV.
