# Supplementary figures and images for: Critical role of deadenylation in regulating poly(A) rhythms and circadian gene expression
Source: PLoS Comput Biol. 2020 Apr 27;16(4):e1007842. doi: 10.1371/journal.pcbi.1007842 (PMC7205317; doi:10.1371/journal.pcbi.1007842)

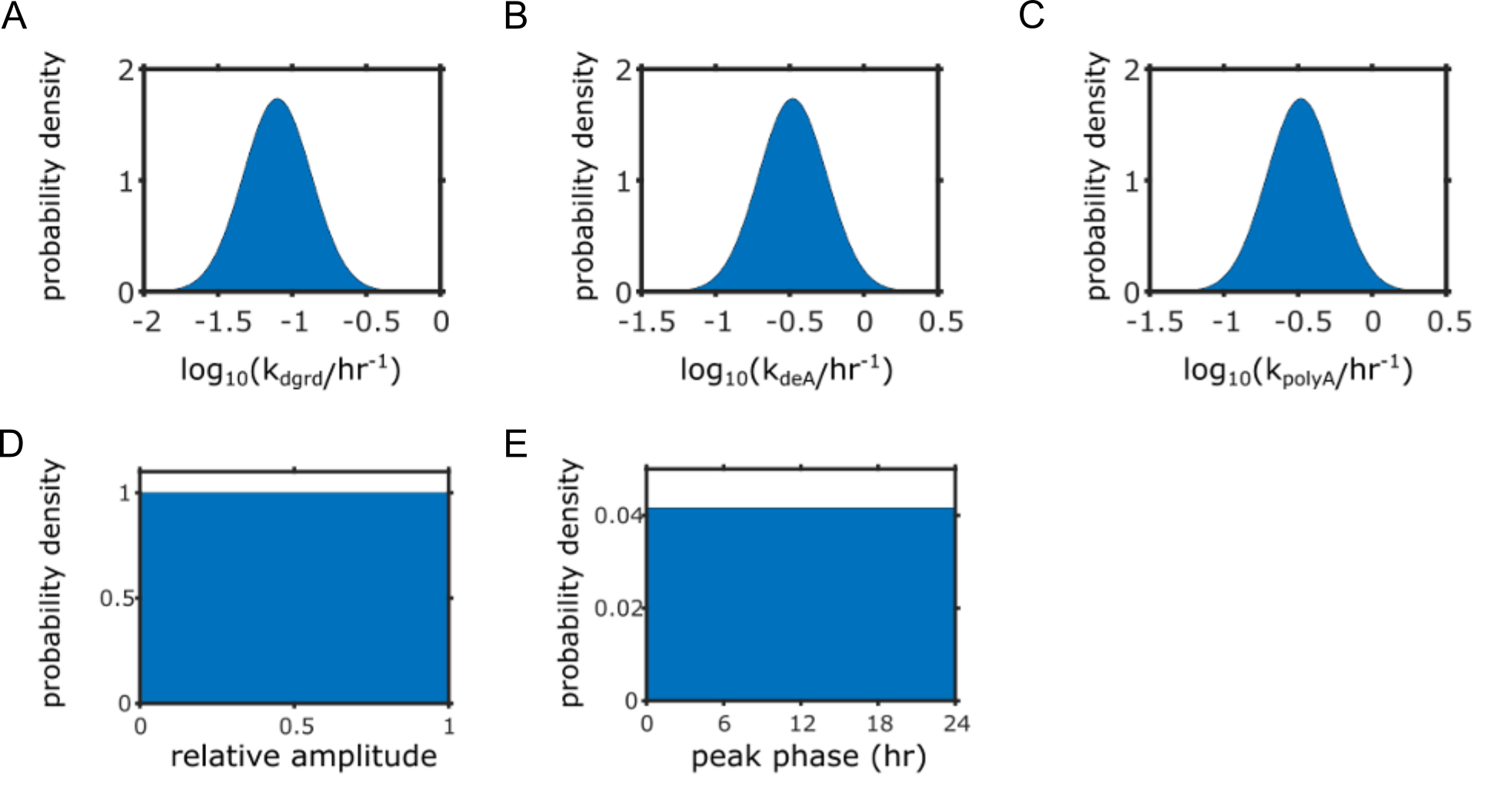

Supplement: S1 Fig — (A) Sampling distribution of mean mRNA degradation rate. (B) Sampling distribution of mean deadenylation rate. (C) Sampling distribution of mean polyadenylation rate. (D) Sampling distribution of relative amplitudes of all rhythmic processes. (E) Sampling distribution of peak phases of all rhythmic processes. (TIF) [file pcbi.1007842.s001.tif]

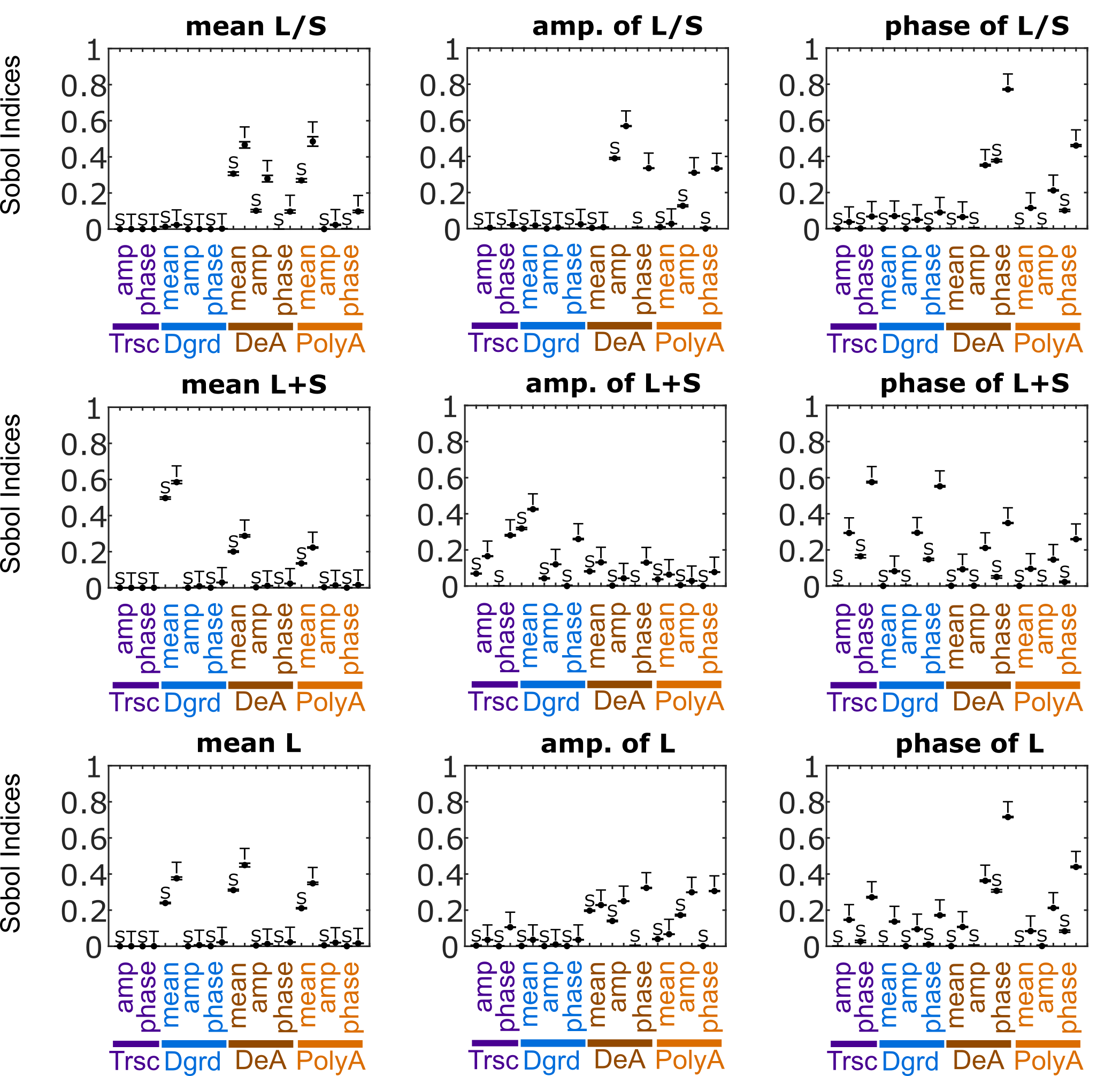

Supplement: S2 Fig — Calculation using Eqs (1) and (2). Label “S” on top: single Sobol indices. Label “T” on top: total Sobol indices. Error bars show the standard deviation of the estimated Sobol indices from 10 repeats. Each repeat was performed using the procedure described in Methods with 𝑁 = 100,000. (TIF) [file pcbi.1007842.s002.tif]

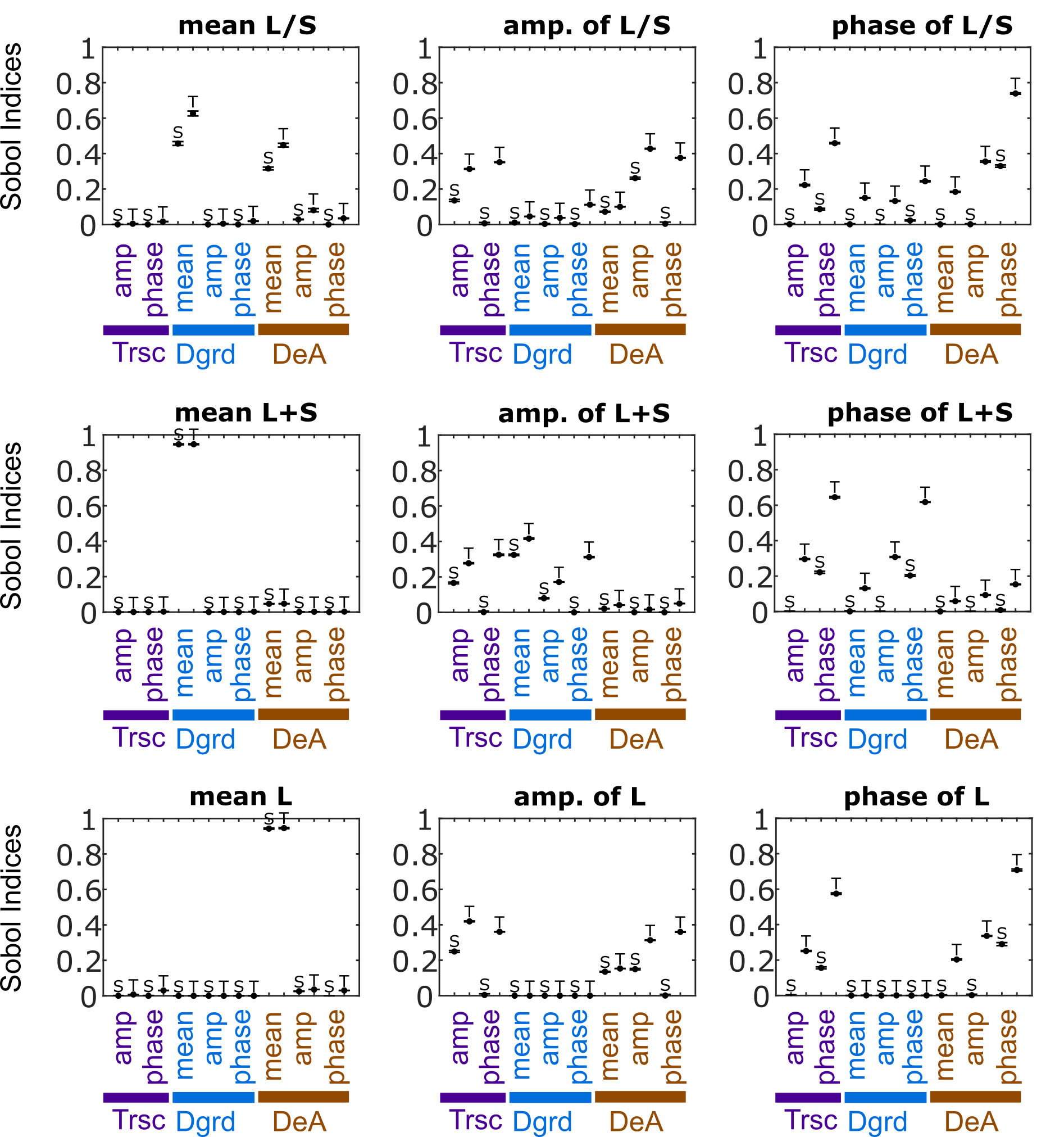

Supplement: S3 Fig — Calculation using Eqs (1) and (2), with 𝜅polyA = 0. Label “S” on top: single Sobol indices. Label “T” on top: total Sobol indices. Error bars show the standard deviation of the estimated Sobol indices from 10 repeats. Each repeat was performed using the procedure described in Methods with 𝑁 = 100,000. (TIF) [file pcbi.1007842.s003.tif]

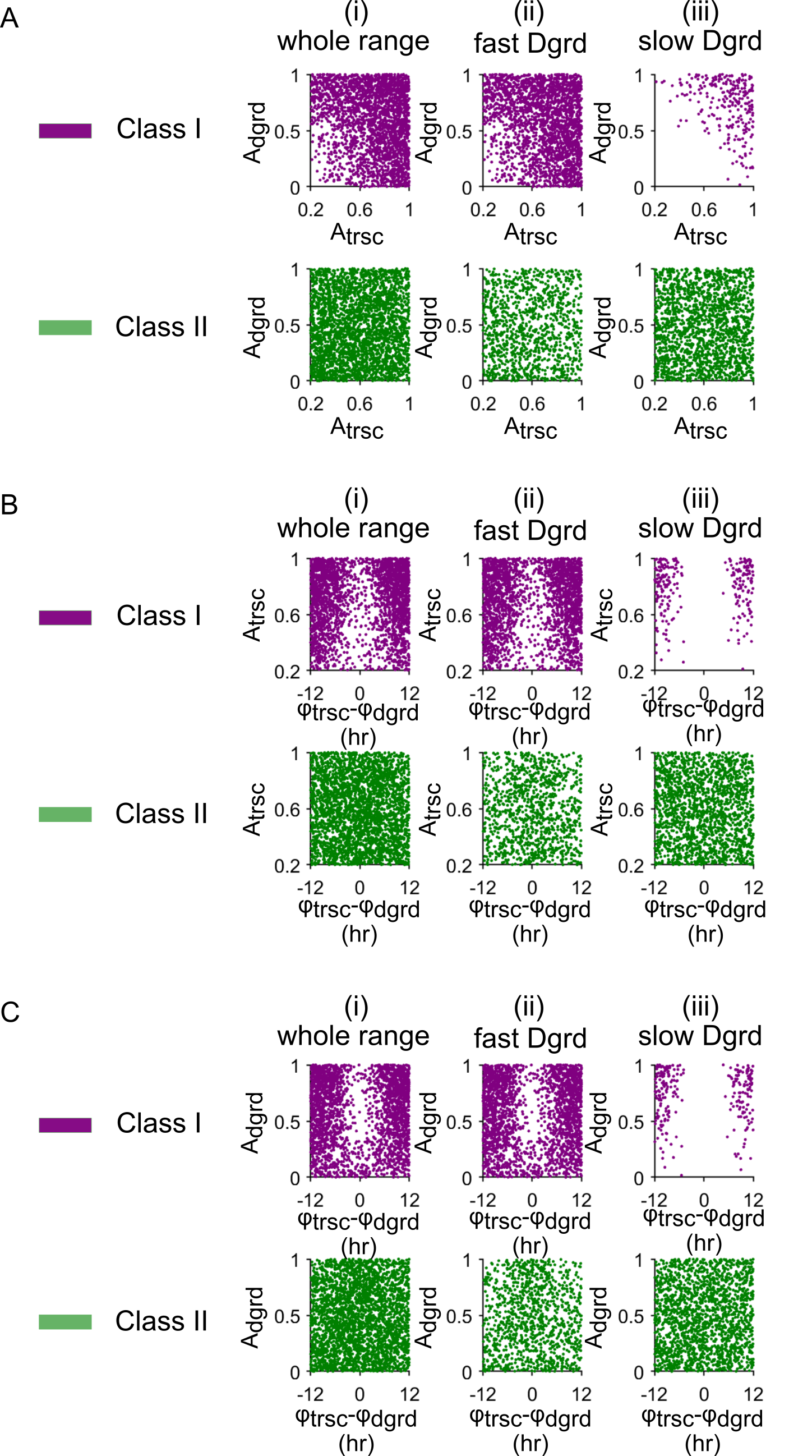

Supplement: S4 Fig — (A) Parameter distributions with respect to the amplitudes of transcription and degradation. (B) Parameter distributions with respect to the phase difference between transcription and degradation and the amplitude of transcription. (C) Parameter distributions with respect to the phase difference between transcription and degradation and the amplitude of degradation. Case (i): Scatter plots for 3,000 Class I sets and 3,000 Class II sets randomly chosen from the 100,000 parameter sets used to produce Fig 4. Case (ii): The parameter sets in case (i) that satisfy −1.15≤log10kdgrd≤0. Case (iii): The parameter sets in case (i) that satisfy −2≤log10kdgrd≤−1.15. As the mean degradation rate, kdgrd, decreases, fewer Class I parameter sets are found in a more confined region. (TIF) [file pcbi.1007842.s004.tif]

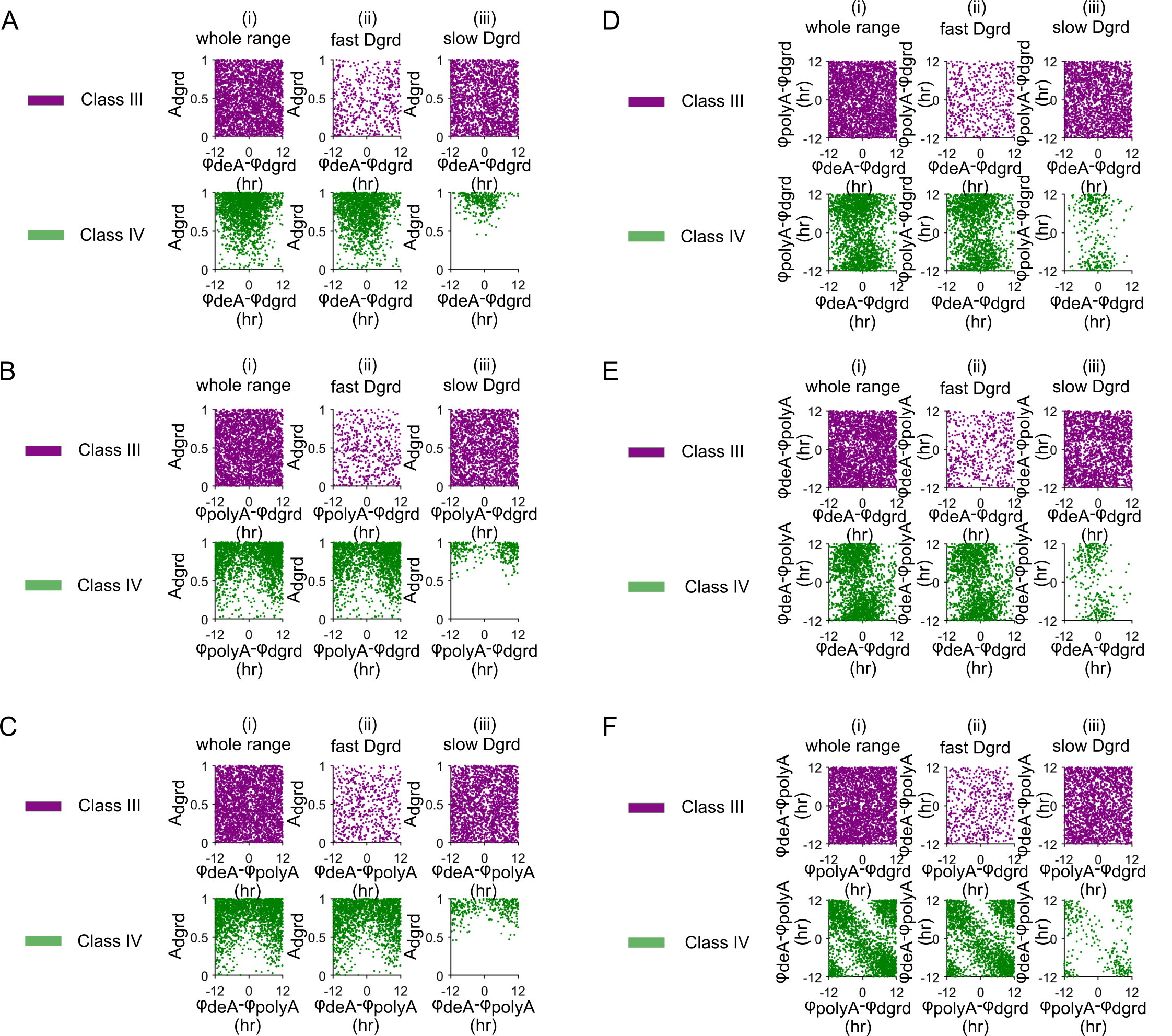

Supplement: S5 Fig — (A-C) Parameter distributions with respect to the degradation amplitude and the phase difference between deadenylation and degradation (A), or between polyadenylation and degradation (B), or between deadenylation and polyadenylation (C). (D-F) Parameter distributions with respect to pairs of phase differences. Case (i): Scatter plots for 3,000 Class III sets and 3,000 Class IV sets randomly chosen from the 100,000 parameter sets used to produce Fig 5. Case (ii): The parameter sets in case (i) that satisfy −1≤log10kdgrd≤1. Case (iii): The parameter sets in case (i) that satisfy −1.5≤log10kdgrd≤−1. As the mean degradation rate, kdgrd, decreases, fewer Class IV parameter sets are found in a more confined region. (TIF) [file pcbi.1007842.s005.tif]

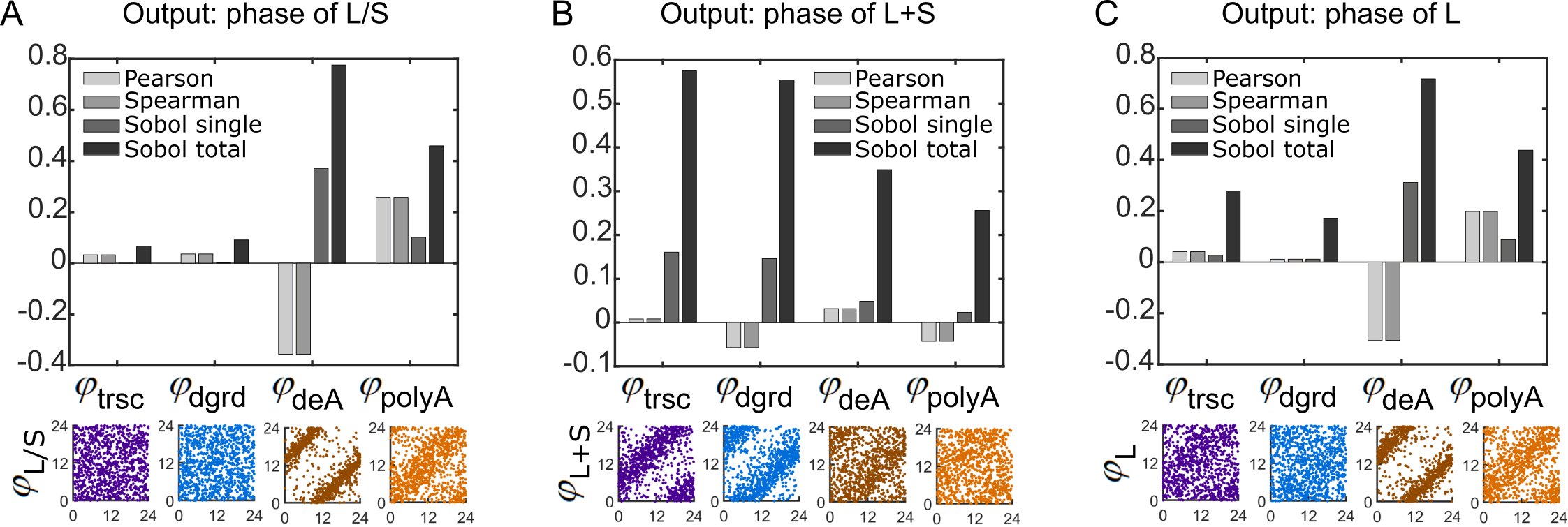

Supplement: S6 Fig — (A) Dependency analyses between the phase of L/S ratio and the phase of each input. (B) Dependency analyses between the phase of L+S and the phase of each input. (C) Dependency analyses between the phase of L and the phase of each input. Scatter plots from Fig 2 for each input-output pair are placed below the corresponding dependency analysis results. (TIF) [file pcbi.1007842.s006.tif]
