# Supplementary material for: Critical role of deadenylation in regulating poly(A) rhythms and circadian gene expression
Source: PLoS Comput Biol. 2020 Apr 27;16(4):e1007842. doi: 10.1371/journal.pcbi.1007842 (PMC7205317; doi:10.1371/journal.pcbi.1007842)
Supplement: S1 File — (PDF) [file pcbi.1007842.s007.pdf]

Setting mean transcription rate as constant does not affect rhythmic pattern

Eqs. (1) and (2) from the Methods section are copied below.

Long-tailed mRNA: $$\frac{dL}{dt} = \kappa_{\mathrm{trsc}}(t) - \kappa_{\mathrm{deA}}(t)L + \kappa_{\mathrm{polyA}}(t)S \quad \text{(S1)}$$

Short-tailed mRNA: $$\frac{dS}{dt} = \kappa_{\mathrm{deA}}(t)L - \kappa_{\mathrm{polyA}}(t)S - \kappa_{\mathrm{dgrd}}(t)S \quad \text{(S2)}$$

Note that $\kappa_{\mathrm{trsc}}(t) = k_{\mathrm{trsc}}\,(1 + A_{\mathrm{trsc}}\cos(\omega(t - \varphi_{\mathrm{trsc}})))$. We can divide both sides of Eqs. (S1) and (S2) by the mean transcription rate constant, k_{trsc} and obtain the following equations:

$$\frac{d\left(L/k_{\mathrm{trsc}}\right)}{dt} = \left(1 + A_{\mathrm{trsc}}\cos\left(\omega t - \varphi_{\mathrm{trsc}}\right)\right) - \kappa_{\mathrm{deA}}(t)\frac{L}{k_{\mathrm{trsc}}} + \kappa_{\mathrm{polyA}}(t)\frac{S}{k_{\mathrm{trsc}}} \quad \text{(S3)}$$

$$\frac{d\left(S/k_{\mathrm{trsc}}\right)}{dt} = \kappa_{\mathrm{deA}}(t)\frac{L}{k_{\mathrm{trsc}}} - \kappa_{\mathrm{polyA}}(t)\frac{S}{k_{\mathrm{trsc}}} - \kappa_{\mathrm{dgrd}}(t)\frac{S}{k_{\mathrm{trsc}}} \quad \text{(S4)}$$

Eqs. (S3) and (S4) show that changing k_{trsc} only causes a strictly proportional change of $L(t)$ and $S(t)$. The rhythmicity patterns, including the peak phases and relative amplitudes, of all output quantities, will not be affected at all. When k_{trsc} changes, the means of $L(t)$ and $S(t)$ change proportionally, but mean L/S ratio remains the same.
