# Supplementary material for: Critical role of deadenylation in regulating poly(A) rhythms and circadian gene expression
Source: PLoS Comput Biol. 2020 Apr 27;16(4):e1007842. doi: 10.1371/journal.pcbi.1007842 (PMC7205317; doi:10.1371/journal.pcbi.1007842)
Supplement: Attachment — Submitted filename: PC_Yao.pdf [file pcbi.1007842.s008.pdf]

The manuscript titled “Critical role of deadenylation in regulating poly(A) rhythms and circadian gene expression” investigates the role of rhythmic deadenylation in regulating circadian gene expression by performing the variance-based global sensitivity analysis. Furthermore, the authors provided the potential mechanism to explain why mRNAs are grouped into three classes based on the presence of rhythms in pre-mRNA and total mRNA. Considering the impact of the result and novelty in the method, I strongly believe that the manuscript fits well with PLOS Computational Biology.

Major comments

1. The interpretation of the results (Line 303~) is not convincing. The current results (Fig. 4) indicate that regardless of the other condition, as long as, mRNA degradation is slow, Class II occurs (note that uniform distribution of Atrsc, Adgrd, ...indicate that Class II do not depend on those). Similarly, Class III appears to the result of just stable mRNA. It is well known that if molecular is stable, it is nearly impossible to generate rhythms even its synthesis/degradation are rhythmic.
2. The method used in the manuscript (Sobol index) would be useful to investigate the other properties of the circadian clock. For instance, it could be useful to identify the hidden crosstalk between clock and disease from rhythmic timeseries data (e.g. while the other approach is used to identify the hidden regulation of PER2 for p53 (Gotoh et al, PNAS (2016), but Sobol index could be more efficient way to do this). Based on phase shift and period/amplitude change caused by clock modulating drugs, Sobol index can be used to find the detailed molecular mechanism how the drug regulates the target clock molecules and source of variations in the drug effect by applying the index to various systems chronopharmacology models (Hirota et al, Science (2012), Kim, Forger et al, CPT:PSP (2013), Kim, Chang et al, Mol Syst Biol (2019)). For instance, PF-670 delays circadian phase by inhibiting CK1d/e and thus PER phosphorylation. Is the delay mainly due to inhibition of PER degradation, nuclear entry of PER, or binding of PER with CRY? It would be nice to

discuss how the sobol index can be applied to understand the circadian clock.

Minor comments

1. More descriptive subtitle would be better instead of “Model”
2. More detailed explanation for Eq. (4) and (5) would be helpful for reader.
3. Fig 1B seems not be referred in the main text.
4. The $(N, 1)$-entry of the matrix $A_B^{(i)}$ in Equation (7) seems $A_{N,1}$ not $A_{N,2}$.
5. Labels (i.e. A, B, C, …) are missing in Figure S1.
6. Is it true that deadenylation is the strongest contributor for S and S/L?
