# Supplementary material for: Critical role of deadenylation in regulating poly(A) rhythms and circadian gene expression
Source: PLoS Comput Biol. 2020 Apr 27;16(4):e1007842. doi: 10.1371/journal.pcbi.1007842 (PMC7205317; doi:10.1371/journal.pcbi.1007842)
Supplement: Attachment — Submitted filename: Yao_2019_circgenexp_response2reviewer.pdf [file pcbi.1007842.s009.pdf]

Detailed List of Responses to Review Comments and Changes in Manuscript

Responses to Reviewer #1:

In this manuscript Yao et al. used mathematical modeling to explore the possible roles of deadenylation in regulating circadian gene expression. I read this work with great interest. It has been an active research area on how circadian rhythm is regulated, and this work shows there is still new mechanism that has not been thoroughly investigated. Through analyzing simple mathematical models that are based on existing experimental observations, these authors convincingly showed that Poly(A) tail length regulation is an important part of circadian gene regulation. I only have a few minor comments for the authors to consider.

Response: We thank the reviewer for taking the time to review our manuscript. We truly appreciate that the reviewer shows great interest in our work and compliments on the significance of our findings.

Minor comments:

1) It took me some efforts to realize that the authors focused on the downstream genes that are regulated by the circadian rhythm network. The authors can make this point more transparent by modifying Fig. 1A. Specifically they can add schematically the circadian rhythm oscillator as an upstream network that affects the four processes, transcription, deadenylation, etc.

Response: We thank the reviewer for pointing out the confusion and suggesting revision to clarify it. Following the reviewer's suggestion, we have modified Fig. 1A to clarify that we focused on clock-controlled genes, rather than the core clock machinery.

A

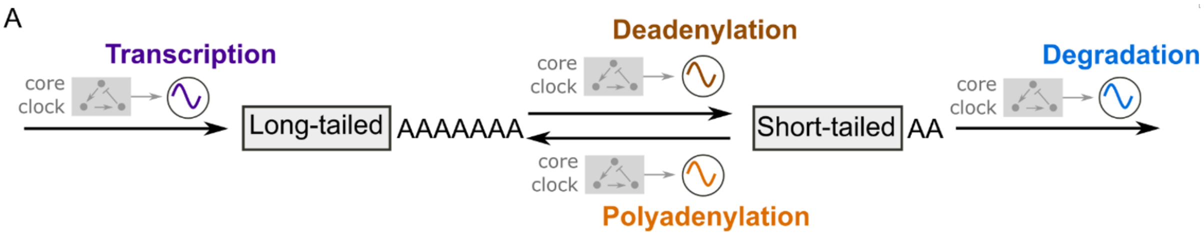

Figure 1. Overview of the study. (A) Schematic diagram of the model. The model describes four processes that control the poly(A) tail length and mRNA abundance: transcription, degradation, cytoplasmic deadenylation and polyadenylation. The rhythmicities of the four processes, i.e., amplitude and phase, are presumably controlled by the core clock mechanism (shaded molecular circuit), which is not explicitly included in the model.

2) Figure 3 is probably the most interesting result of this work. I notice the phase plots and some polar coordinate numbers overlap in the figures. The authors may adjust the plotting.

Response: Figure 3 has been modified as suggested.

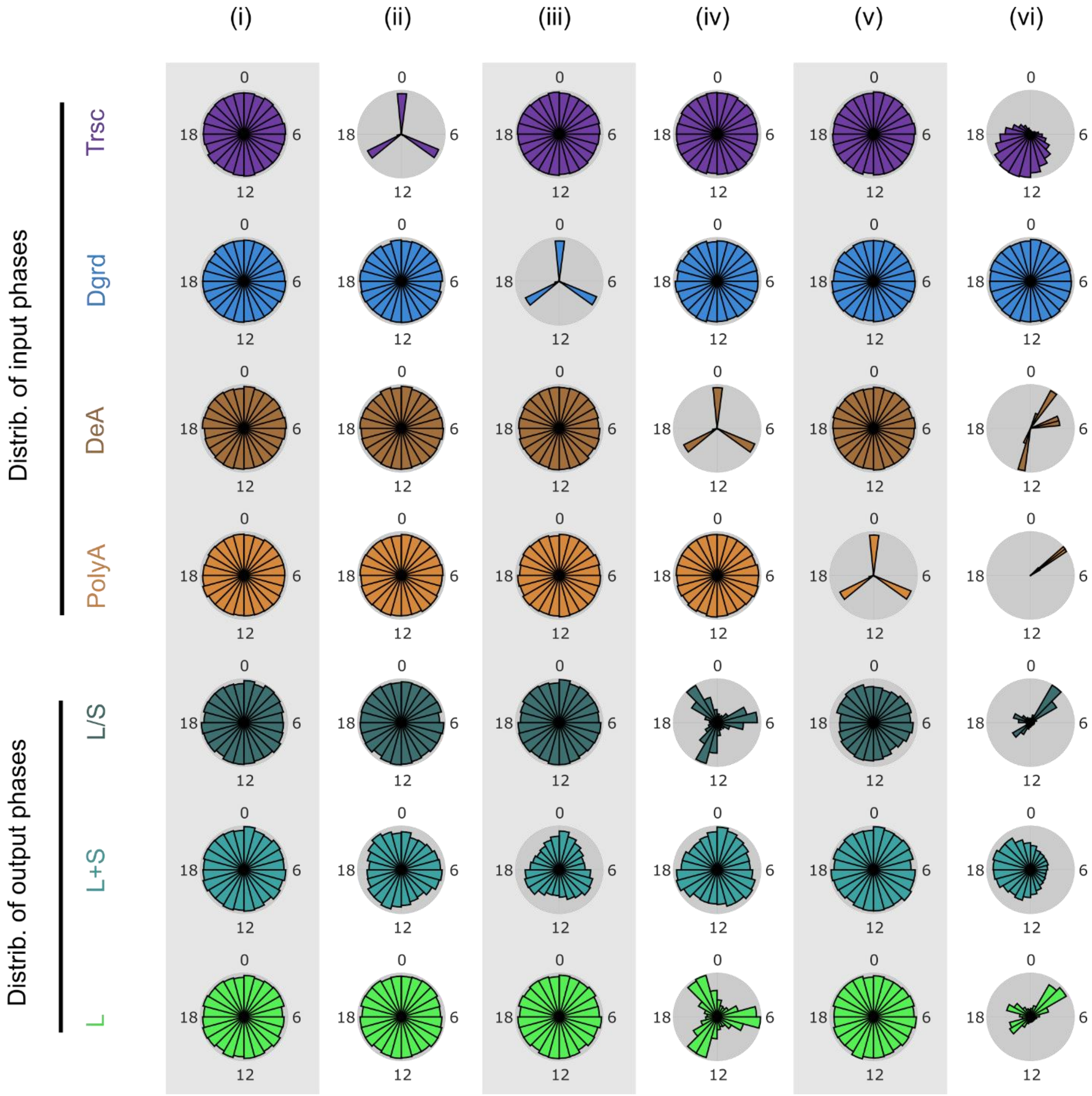

Figure 3.

3) It is impressive that some rich information can already deduced from the model given by equations 1 and 2. It is justified to start with such simple models, which are convenient for thorough parameter space examination. There are, however, certain limitations on coarse-graining the poly-A tail length as two discrete states. Mathematically one can treat the process as a reaction-diffusion process, and can write down either a discrete chemical master equation or continuous 1D process (described by Langevin equations or Fokker-Planck equations). The authors may discuss limitation of their formulation and alternative future studies.

Response: We thank the reviewer for pointing out the limitation of coarse-graining the poly(A) tail length as two states and suggesting future study. In the revised manuscript, we have added the descriptions of this limitation and potential future study on p. 21:

"In our current model, the poly(A) regulation has been coarse-grained as one-step conversions between a long-tailed and a short-tailed mRNA subpopulation. Such coarse-graining retains the most essential kinetic features of the poly(A) regulation processes, while allowing for significant reduction of the model and efficient global parameter sensitivity analysis. From such analysis we identified the critical role of deadenylation in rhythmic regulation. In reality, both deadenylation and polyadenylation act sequentially, i.e., adding or subtracting one adenosine at a time. Unlike one-step chemical reactions, the kinetics of sequential processes are often non-exponential (Deneke et al., 2013; Eisen et al., 2019). To evaluate the rhythmicities of poly(A) regulation and gene expression more accurately, we will include a linear reaction chain in the model to account for sequential steps of deadenylation and polyadenylation in our future work."

Responses to Reviewer #2:

The manuscript titled "Critical role of deadenylation in regulating poly(A) rhythms and circadian gene expression" investigates the role of rhythmic deadenylation in regulating circadian gene expression by performing the variance-based global sensitivity analysis. Furthermore, the authors provided the potential mechanism to explain why mRNAs are grouped into three classes based on the presence of rhythms in pre-mRNA and total mRNA. Considering the impact of the result and novelty in the method, I strongly believe that the manuscript fits well with PLOS Computational Biology.

Response: We thank the reviewer for taking the time to review our manuscript and for the valuable and constructive comments and suggestions. We truly appreciate that the reviewer points out the significance and novelty of our work, as well as its fit with PLOS Computational Biology.

Major comments:

1. The interpretation of the results (Line 303~) is not convincing. The current results (Fig. 4) indicate that regardless of the other condition, as long as, mRNA degradation is slow, Class II occurs (note that uniform distribution of Atrsc, Adgrd, …. indicate that Class II do not depend on those). Similarly, Class III appears to the result of just stable mRNA. It is well known that if molecular is stable, it is nearly impossible to generate rhythms even its synthesis/degradation are rhythmic.

Response: We fully agree with the reviewer that slow mRNA degradation tends to result in Class II. However, degradation rate is not the only factor distinguishing between Class I and Class II, as the distribution of degradation rate significantly overlap between the two classes (Fig. 4C). For degradation rates that are shared by both classes, other factors, such as the phase difference between transcription and degradation (Fig. 4D), and the amplitudes of transcription and degradation (Fig. 4E-F), also play significant roles.

Then one might ask, why are Class I parameter sets significantly enriched in certain ranges for the factors in Fig. 4D-F, whereas Class II parameter sets show nearly even distributions in those factors? This question can be more easily understood by "reconstructing" the parameter distributions in the global parameter space from the 1D distributions (Fig. R1 below).

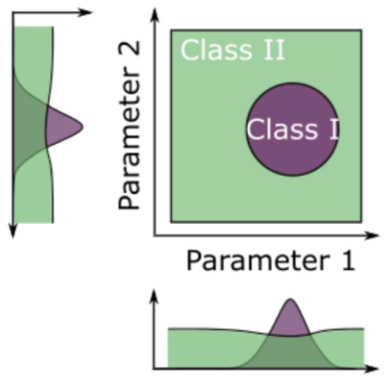

Figure R1: Illustrative diagram for parameter regions in global parameter space. Class I parameter sets (purple) are distributed in a confined region in the global parameter space, which is projected as narrow distributions in all single-parameter subspaces. Class II parameter sets (green) are distributed in the broad area outside the Class I region, which is projected as nearly even distributions in single-parameter subspaces.

In the global parameter space, the Class I parameter sets are distributed in a confined region (Fig. R1, purple), whereas Class II parameter sets are broadly distributed in the remaining region not taken by Class I. Because the Class II region is broad, the 1D projections of this region appear nearly even. This led to our original interpretation of Fig. 4D-F (currently on p. 16): "generation of significant rhythmicity in L+S (Class I) requires sufficient phase difference between transcription and degradation, and sufficiently high amplitudes of transcription and degradation, simultaneously. If any of these conditions are not satisfied, total mRNA abundance would not have significant rhythmicity (Class II)." From an alternative perspective, the three factors in Fig. 4D-F essentially compensate each other in producing "no rhythm" in total mRNA. A similar argument applies to Class III/IV for the interpretation of Fig. 5D, E.

To clarify this point in the manuscript, we have plotted the pairwise 2D distributions of Class I-IV parameter sets as Figs. S4 (Class I and II) and S5 (Class III and IV). Figs. S4 and S5 show confined distributions of Class I and Class IV parameter sets, compared to Class II and Class III, respectively. Notably, the Class I (Fig. S4) and Class IV (Fig. S5) regions shrink as mRNA degradation rate decreases, which is consistent with the prediction that slow mRNA degradation rate disfavors Class I (Fig. 4C) and Class IV (Fig. 5C). Because the global parameter space has much more than two dimensions, 2D projections (Figs. S4 and S5) cannot effectively visualize a broad area with a hole in the high-dimensional space, like the green area illustrated in Fig. R1 for Class II (and similarly for Class III). Nevertheless, Figs. S4B(ii) and C(ii) do show a weak reduction of Class II parameter sets in the region where Class I is strongly concentrated, which is consistent with the idea illustrated in Fig. R1.

Figs. S4 and S5 are cited on ln. 331 and ln. 377 in the manuscript.

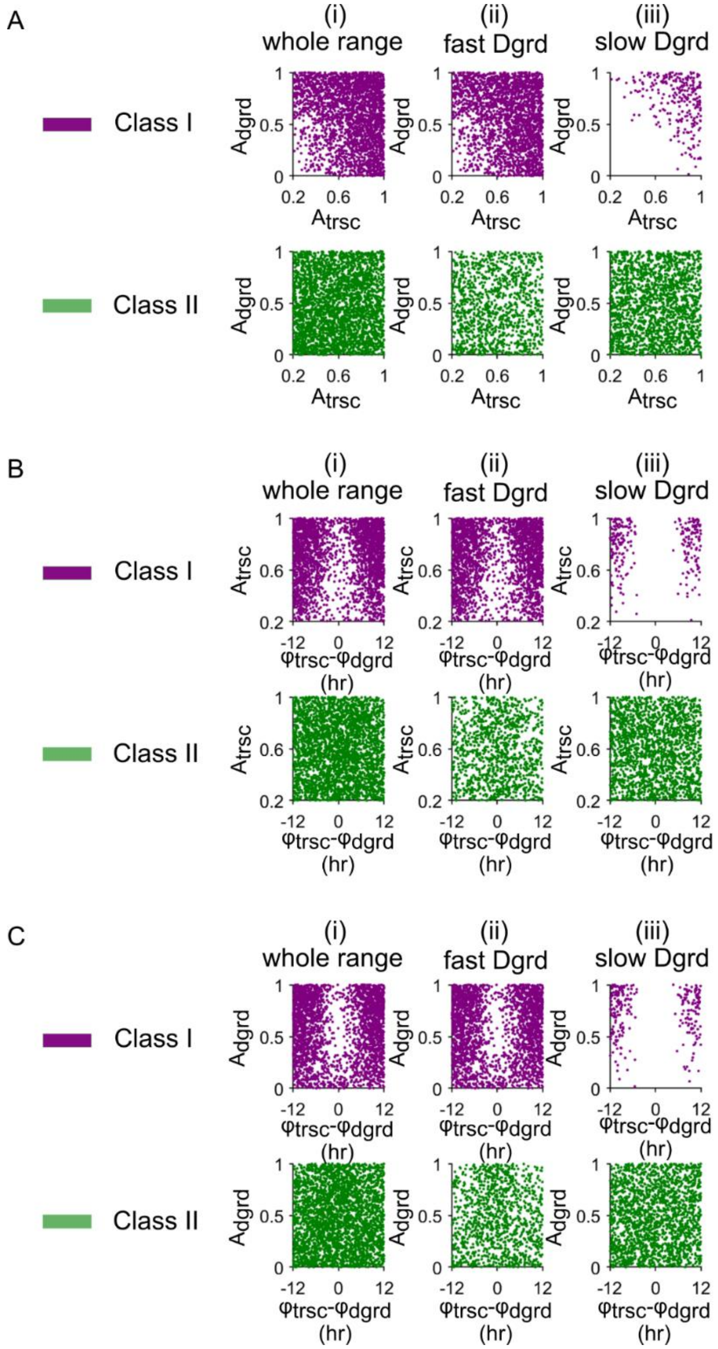

Figure S4. 2D distributions of Class I and Class II parameter sets show that the Class I parameter sets are found in a more confined region than the Class II parameter sets. (A) Parameter distributions on 2D space combining the amplitudes of transcription and degradation. (B) Parameter distributions on 2D space combining the phase difference between transcription and degradation and the amplitude of transcription. (C) Parameter distributions on 2D space combining the phase difference between transcription and degradation and the amplitude of degradation. Case (i): Scatter plots for 3,000 Class I sets and 3,000 Class II sets randomly chosen from the 100,000 parameter sets used to produce Fig. 4. Case (ii): The parameter sets in case (i) that satisfy $-1.15 \leq \log_{10} k_{\mathrm{dgrd}} \leq 0$. Case (iii): The parameter sets in case (i) that satisfy $-2 \leq \log_{10} k_{\mathrm{dgrd}} \leq -1.15$. As the mean degradation rate, k_{dgrd}, decreases, there are fewer Class I parameter sets, and they are distributed in a more confined region.

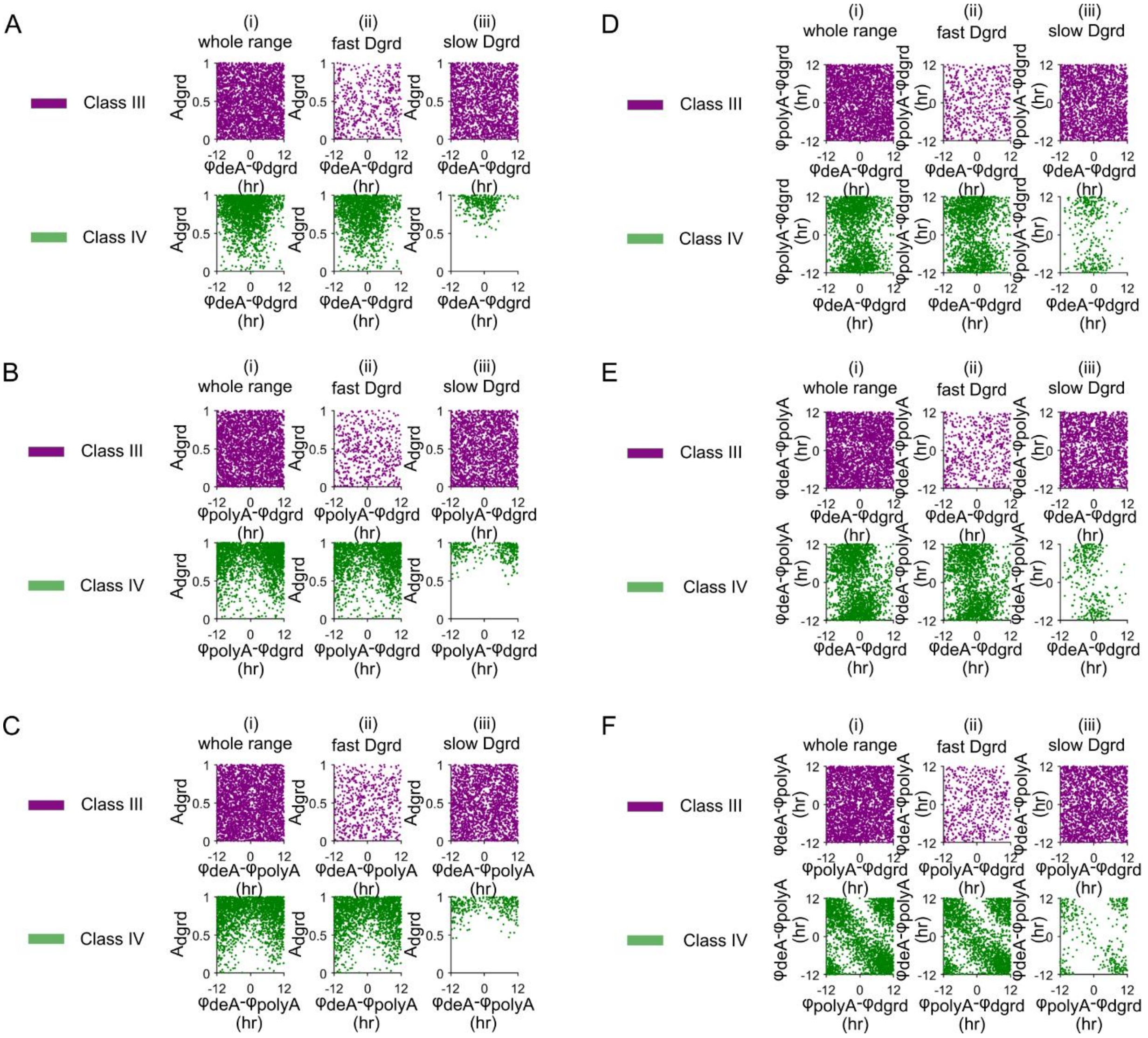

Figure S5. 2D distributions of Class III and Class IV parameter sets show that the Class IV parameter sets are found in a more confined region than Class III parameter sets. (A-C) Parameter distributions on 2D space combining the degradation amplitude and the phase difference between deadenylation and degradation (A), or between polyadenylation and degradation (B), or between deadenylation and polyadenylation (C). (D-F) Parameter distributions on 2D space combining pairs of phase differences. Case (i): Scatter plots for 3,000 Class III sets and 3,000 Class IV sets randomly chosen from the 100,000 parameter sets used to produce Fig. 5. Case (ii): The parameter sets in case (i) that satisfy $-1 \leq \log_{10} k_{\mathrm{dgrd}} \leq 1$. Case (iii): The parameter sets in case (i) that satisfy $-1.5 \leq \log_{10} k_{\mathrm{dgrd}} \leq -1$. As the mean degradation rate, k_{dgrd}, decreases, there are fewer Class IV parameter sets, and they are distributed in a more confined region.

2. The method used in the manuscript (Sobol index) would be useful to investigate the other properties of the circadian clock. For instance, it could be useful to identify the hidden crosstalk

between clock and disease from rhythmic time series data (e.g. while the other approach is used to identify the hidden regulation of PER2 for p53 (Gotoh et al, PNAS (2016), but Sobol index could be more efficient way to do this). Based on phase shift and period/amplitude change caused by clock modulating drugs, Sobol index can be used to find the detailed molecular mechanism how the drug regulates the target clock molecules and source of variations in the drug effect by applying the index to various systems chronopharmacology models (Hirota et al, Science (2012)). For instance, PF-670 delays circadian phase by inhibiting CK1d/e and thus PER phosphorylation. Is the delay mainly due to inhibition of PER degradation, nuclear entry of PER, or binding of PER with CRY? It would be nice to discuss how the sobol index can be applied to understand the circadian clock.

Response: We thank the reviewer for pointing out the transferability of the Sobol's method to other chronobiology studies. We have added a discussion about the advantage of the Sobol's method in analyzing chronobiology models on pp. 22-23:

"In addition, the Sobol's method serves as a particularly powerful tool for parameter sensitivity analysis for models in chronobiology. In chronobiology models, oscillation phases are often important quantities of interest. As circular variables, i.e., ZT 0 = ZT 24, phases are intrinsically nonlinear and non-monotonic. Analyzing nonlinear and non-monotonic variables using classic correlation and dependency analyses, such as Pearson correlation and Spearman correlation, could lead to misleading conclusions, because these methods are based on assumptions about linear and monotonic relations between the analyzed data. For example, in our model, Pearson and Spearman correlation analyses demonstrate strong negative correlation between the phases of deadenylation and L/S ratio, a spurious conclusion due to the circular nature of phases (**Fig. S6**); other pairs of input and output phases suffer different levels of distortion in their Pearson and Spearman correlations (**Fig. S6**). Based on variance decomposition (see Methods), the Sobol's method circumvents these problems and can effectively analyze nonlinear and non-monotonic variables (Iooss and Lemaitre, 2015). The method can be used widely in chronobiology models to identify the key factors that drive phases of target quantities, such as the phase difference between PER2 and TP53, whose interaction is critical for the crosstalk between the circadian clock and cell cycle (Gotoh et al., 2016). Furthermore, the Sobol's method would be useful in model-driven chronopharmacology research (Dallmann et al., 2014; Hirota et al., 2012; Kim et al., 2013), a particularly exciting new area, to elucidate the molecular mechanism of the therapy or drug and the source of variations in the therapeutic effect."

We also added Fig. S6 to compare the Sobol's method with Pearson and Spearman analyses, using our model as an example.

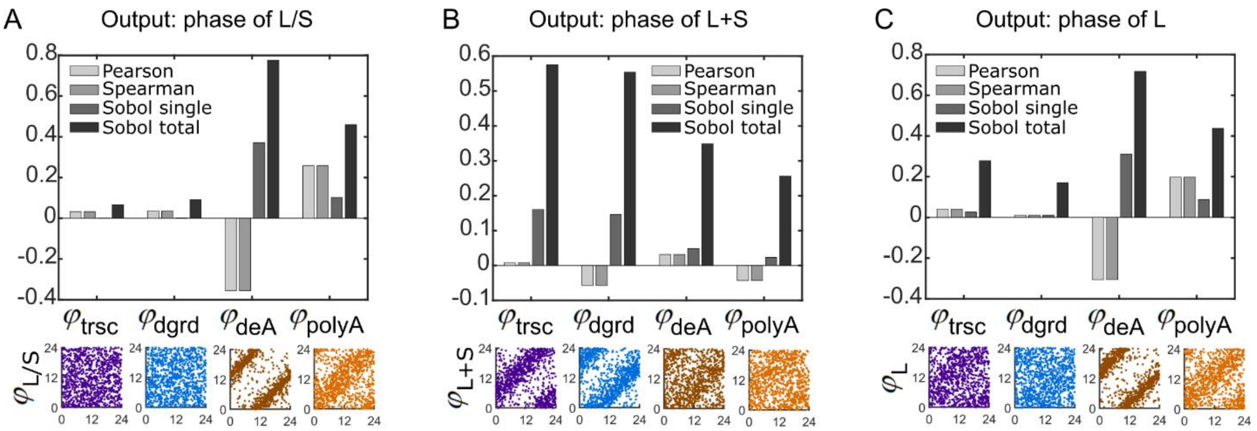

Figure S6. Comparison among Pearson correlation, Spearman correlation and Sobol indices. (A) Dependency analyses between the phase of L/S ratio and the phase of each input. (B) Dependency analyses between the phase of L+S and the phase of each input. (C) Dependency analyses between the phase of L and the phase of each input. Scatter plots from Fig. 2 for each input-output pair are placed below the corresponding groups of results.

Minor comments

1. More descriptive subtitle would be better instead of "Model"

Response: The subtitle has been changed to "Model for rhythmic mRNA and poly(A) tail regulation" on p. 6.

2. More detailed explanation for Eq. (4) and (5) would be helpful for reader.

Response: The Sobol's method is now explained in more details on pp. 26-27 to facilitate readers' understanding. With one equation added to illustrate the conceptual basis of the Sobol's method, Eqs. (4) and (5) have become Eqs. (5) and (6). Text quoted from pp. 26-27:

"To evaluate the impact of each model parameter (e.g., phase of deadenylation) on each model output (e.g., relative amplitude of L/S ratio), we used a variance-based global parameter sensitivity analysis method, the Sobol indices (Iooss and Lemaitre, 2015; Sobol, 2001). The conceptual basis of this method is functional decomposition of the variance of an output Y into contributions from each parameter and interactions between parameters (Eq. (1)).

$$\mathrm{Var}(Y) = \sum_i V_i(Y) + \sum_{i<j} V_{ij}(Y) + \cdots + V_{1,2,\ldots,k}(Y) \tag{1}$$

In Eq. (1), $V_i(Y) = \mathrm{Var}_{X_i}\left(\mathrm{E}_{X_{\sim i}}(Y \mid X_i)\right)$ is the contribution from the i-th parameter alone. Here $X_{\sim i}$ denotes the combined parameter set except for the i-th parameter. $\mathrm{E}_{X_{\sim i}}(Y \mid X_i)$ denotes the expectation of output Y conditional on a fixed value for X_i (while the other parameters randomly vary). $\mathrm{Var}_{X_i}\left(\mathrm{E}_{X_{\sim i}}(Y \mid X_i)\right)$ then denotes the variance of the calculated conditional

expectation as X_i varies (Eq.(2)). The second term of Eq.(1), $V_{ij}(Y) = \mathrm{Var}_{X_i,X_j}\left(\mathrm{E}_{X_{\sim ij}}\left(Y \mid X_i, X_j\right)\right) - V_i(Y) - V_j(Y)$, is the contribution from the interactions between the i-th and j-th parameters, where $X_{\sim ij}$ denotes the combined parameter set except for the i-th and j-th parameters. Contributions from higher-order interactions between parameters are defined similarly as $V_{ij}(Y)$.

The Sobol indices are then defined as fractions of the decomposed terms in Eq.(2) out of the total variance, $\mathrm{Var}(Y)$. In practice, only the single (Eq.(2)) and total-effect (Eq.(3)) indices are calculated because relatively simple algorithm as described below can be designed. Specifically, the single Sobol index, S_i, characterizes the contribution of variance in X_i alone to the total variance in Y (Eq.(2)). The total-effect, or total index, S_{Ti}, characterizes the contribution of variance in X_i, as well as the variance caused by its coupling with other parameters, to the total variance in Y (Eq.(3)). Conveniently, the total-effect contribution equals $\mathrm{E}_{X_{\sim i}}\left(\mathrm{Var}_{X_i}\left(Y \mid X_{\sim i}\right)\right)$. Here $\mathrm{Var}_{X_i}\left(Y \mid X_{\sim i}\right)$ denotes the variance of output Y conditional on a fixed set of $X_{\sim i}$ (while X_i randomly varies). $\mathrm{E}_{X_{\sim i}}\left(\mathrm{Var}_{X_i}\left(Y \mid X_{\sim i}\right)\right)$ then denotes the expectation of the calculated variance as $X_{\sim i}$ varies (Eq.(3)). The larger the single and total indices are, the more sensitive Y is to X_i, or the more impact X_i has on Y.

$$S_i = \frac{\mathrm{Var}_{X_i}\left(\mathrm{E}_{X_{\sim i}}\left(Y \mid X_i\right)\right)}{\mathrm{Var}(Y)} \tag{2}$$

$$S_{Ti} = \frac{V_i + \sum_{j \neq i} V_{ij} + \sum_{j \neq i, k \neq i, j<k} V_{ijk} + \cdots}{\mathrm{Var}(Y)} = \frac{\mathrm{E}_{X_{\sim i}}\left(\mathrm{Var}_{X_i}\left(Y \mid X_{\sim i}\right)\right)}{\mathrm{Var}(Y)} \tag{3}$$

”

3. Fig 1B seems not be referred in the main text.

Response: Fig 1B is now referred to on ln. 131, 178, 193, 590 and 594.

4. The (N, 1)-entry of the matrix $A_B^{(i)}$ in Equation (7) seems $A_{N,1}$ not $A_{N,2}$.

Response: The wrong subscript has been corrected.

5. Labels (i.e. A, B, C, …) are missing in Figure S1.

Response: Panel labels have been added in Figure S1.

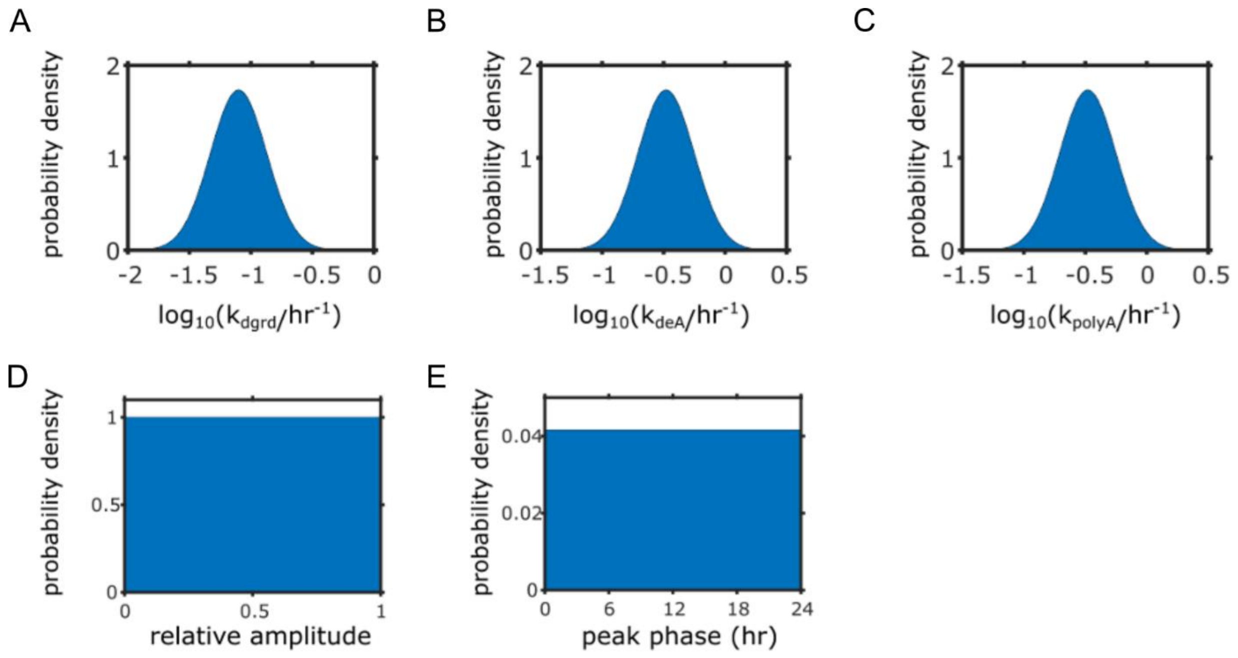

Figure S1. Sampling distributions of the model parameters. (A) Sampling distribution of mean mRNA degradation rate. (B) Sampling distribution of mean deadenylation rate. (C) Sampling distribution of mean polyadenylation rate. (D) Sampling distribution of relative amplitudes of all rhythmic processes. (E) Sampling distribution of peak phases of all rhythmic processes.

6. Is it true that deadenylation is the strongest contributor for S and S/L?

Response: Yes, deadenylation is the strongest contributor for rhythmicities in S and S/L. We have calculated the Sobol indices for S and S/L (Fig. R2 below). The amplitudes and phases of the two quantities strongly depend on the rhythmicities of deadenylation and polyadenylation, similar to L and L/S. Note that S/L is just the reciprocal of L/S, Hence, the corresponding Sobol indices for S/L are expectedly almost identical to those for L/S.

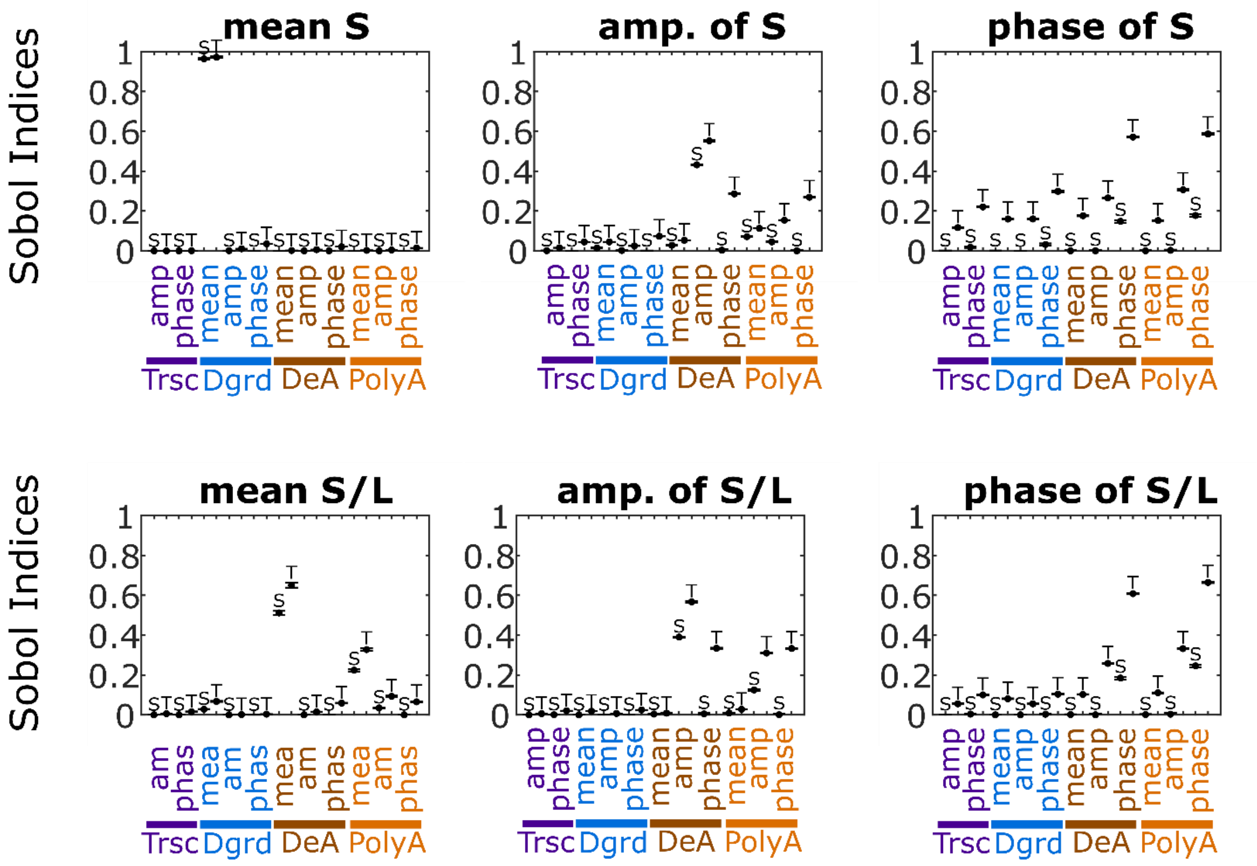

Figure R2: Sobol indices for outputs, S and S/L.

Interestingly, the mean level of S is almost exclusively determined by the mean degradation rate (Fig. R2). This can be understood from the steady-state level of S when all four input processes are non-rhythmic. In this non-rhythmic case, $S_{ss} = k_{\mathrm{trsc}}/k_{\mathrm{dgrd}}$ only depends on the rate constants for transcription and degradation. Hence, in the rhythmic model, the mean S is expected to mainly depend on the mean rates of transcription and degradation. Because the mean transcription rate is set to be 1 in our study (as it does not affect the rhythmic patterns in any case), the mean S strongly depends only on the mean degradation rate. Following a similar reasoning, mean L depends on mean deadenylation and polyadenylation rates (Fig. S2), because in the non-rhythmic case, $L_{ss} = k_{\mathrm{trsc}}(k_{\mathrm{polyA}} + k_{\mathrm{dgrd}})/k_{\mathrm{deA}}k_{\mathrm{dgrd}}$ depends on k_{polyA} and k_{deA}. However, in the model without cytoplasmic polyadenylation ($k_{\mathrm{polyA}} = 0$), L depends only on mean deadenylation rate (Fig. S3), because the corresponding non-rhythmic case has $L_{ss} = k_{\mathrm{trsc}}/k_{\mathrm{deA}}$.

Responses to Reviewer #3:

The authors address a hot topic – the differential regulation of clock-controlled genes (CCGs). It has been found earlier (Naef, Westermark, Robles, Koike, Heyd …) that different stages of gene products display differential rhythmicity. Kojima et al. 2012 provided a rich resource of rhythmic genes associated with mRNA polyadenylation. In the submitted manuscript a theoretical framework is provided to connect different processes to measured quantities.

The paper is clearly written and the selected model type is consistent with the sparse experimental data. Technically speaking, linear non-autonomous differential equations are simulated for 11 randomly selected parameters. Scatter plots and global sensitivity analysis help to quantify the dominant regulations. The theoretical findings are connected to observations (phases of CCGs, phases of deadenylases). Below I list some suggestion to improve the already very good manuscript.

Response: We thank the reviewer for his/her time and for the very positive review of our work. We truly appreciate his/her valuable and constructive comments and suggestions.

Minor comments:

1. I am aware that redundancy is helpful but abstract, summary, introduction, discussion are quite redundant. Practically identical sentences should be avoided. In the Author Summary a different level of abstraction compared to the abstract might help readers.

Response: We agree that there was a substantial overlap in the Author Summary, Introduction and Discussion, and therefore revised these sections to simplify and/or remove redundancy. Because the changes are scattered in many places, we do not paste all the changes here. Please refer to the Track Change version for the detailed changes.

2. Regarding posttranscriptional circadian regulations some more references might be of interest: F. Heyd regarding splicing, M. Young, M. Brunner and K. Vanselow regarding nuclear import/export).

Response: These references have been added on p. 4.

3. K. Thurley (PNAS 2017) developed a general theory of rhythmic fluxes along chains and introduced circadian response analysis (CRA). These results are highly relevant to interpret the model in Figure 1.

Response: We now refer to the interesting paper and CRA in the discussion on p. 19.

"These four processes jointly regulate the circadian gene expression driven by the core clock (**Fig. 1A**), with a principle similar to a previous theoretical study that investigates rhythmic fluxes along metabolic chains using circadian response analysis (Thurley et al., 2017)."

4. Page 5: …recently …. 2012???

Response: The word "recently" has been removed on ln. 80. It has also been removed or replaced on ln. 20 and 117, when referring to the same paper.

5. Linear systems as in Equations 1-3 can be treated analytically using e.g. variation of constants (see also Luck & Westermark and Thurley 2017). I am aware that the resulting expressions with 4 periodic terms become very complicated. Anyway, the authors should discuss why they prefer numerical solutions.

Response: We chose numerical solutions, because approximate analytical solutions were too complex and intractable, as the reviewer suspected. At the beginning, we did use variation of constants to find the approximate analytic solution for $L(t)$ and $S(t)$. Specifically, we plugged the following ansatzes into Eqs. (1) and (2), and solved for the coefficients.

$$L(t) \approx A_0 + A_1 \cos(\omega t) + B_1 \sin(\omega t)$$

$$S(t) \approx C_0 + C_1 \cos(\omega t) + D_1 \sin(\omega t)$$

Unfortunately, the analytical solutions are rather complex and intractable. For example, the peak phase of long-tailed mRNA,

$$\varphi_L = \begin{cases} \dfrac{24\text{ h}}{2\pi} \arctan\left(\dfrac{A_1}{B_1}\right), & \text{if } \dfrac{A_1}{B_1} \geq 0 \\ \dfrac{24\text{ h}}{2\pi}\left(\arctan\left(\dfrac{A_1}{B_1}\right) + \pi\right), & \text{if } \dfrac{A_1}{B_1} < 0 \end{cases}$$

where A_1 equals

```
4*AdeA^2*Atrsc*KdeA*Kdgrd^3 - 16*Atrsc*KdeA*Kdgrd^3 + 8*Adgrd^2*Atrsc*KdeA*Kdgrd^3 + 16*AdeA*KdeA*Kdgrd^3*cos(PdeA) + 16*AdeA*Kdgrd*Omega^3*sin(PdeA) + 16*AdeA*Kdgrd^3*Omega*sin(PdeA) +
16*AdeA*KpolyA*Omega^3*sin(PdeA) - 16*ApolyA*KpolyA*Omega^3*sin(PpolyA) + 16*AdeA*KpolyA^2*Omega^2*cos(PdeA) - 16*ApolyA*KpolyA^2*Omega^2*cos(PpolyA) - 16*Atrsc*KdeA*Kdgrd^2*KpolyA -
16*Atrsc*KdeA*Kdgrd*Omega^2 - 4*AdeA^2*Atrsc*KdeA*Kdgrd^3*cos(2*PdeA) - 4*AdeA*ApolyA^2*KpolyA^2*Omega^2*cos(PdeA) + 4*AdeA^2*Atrsc*KdeA*Kdgrd^2*KpolyA + 4*Adgrd^2*Atrsc*KdeA*Kdgrd^2*KpolyA +
4*AdeA^2*Atrsc*KdeA*Kdgrd*Omega^2 + 16*AdeA*KdeA*Kdgrd^2*KpolyA*cos(PdeA) + 16*Adgrd*KdeA*Kdgrd^2*KpolyA*cos(Pdgrd) - 16*ApolyA*KdeA*Kdgrd^2*KpolyA*cos(PpolyA) + 16*AdeA*KdeA*Kdgrd*Omega^2*cos(PdeA) +
16*AdeA*KdeA*KpolyA*Omega^2*cos(PdeA) + 16*AdeA*Kdgrd*KpolyA*Omega^2*cos(PdeA) - 16*Adgrd*Kdgrd*KpolyA*Omega^2*cos(Pdgrd) - 16*ApolyA*KdeA*KpolyA*Omega^2*cos(PpolyA) +
16*AdeA*Kdgrd*KpolyA^2*Omega*sin(PdeA) + 32*AdeA*Kdgrd^2*KpolyA*Omega*sin(PdeA) + 16*Adgrd*Kdgrd*KpolyA^2*Omega*sin(Pdgrd) + 16*Adgrd*Kdgrd^2*KpolyA*Omega*sin(Pdgrd) -
16*ApolyA*Kdgrd*KpolyA^2*Omega*sin(PpolyA) - 16*ApolyA*Kdgrd^2*KpolyA*Omega*sin(PpolyA) - 2*AdeA^2*Adgrd^2*Atrsc*KdeA*Kdgrd^3 - 8*AdeA*Adgrd^2*KdeA*Kdgrd^3*cos(PdeA) -
8*AdeA*Adgrd^2*Kdgrd^3*Omega*sin(PdeA) - AdeA^2*Adgrd^2*Atrsc*KdeA*Kdgrd^2*KpolyA - 4*AdeA^2*Adgrd*KdeA*Kdgrd^2*KpolyA*cos(Pdgrd) + 4*AdeA^2*ApolyA*KdeA*Kdgrd^2*KpolyA*cos(PpolyA) +
4*AdeA^2*ApolyA*KdeA*KpolyA*Omega^2*cos(PpolyA) - 4*AdeA*Adgrd^2*Kdgrd^2*KpolyA*Omega*sin(PdeA) - 4*AdeA*ApolyA^2*Kdgrd*KpolyA^2*Omega*sin(PdeA) + 4*AdeA*ApolyA^2*KpolyA^2*Omega^2*cos(2*PpolyA)*cos(PdeA)
+ 4*AdeA*ApolyA^2*KpolyA^2*Omega^2*sin(2*PpolyA)*sin(PdeA) + 16*Adgrd*KdeA*Kdgrd*KpolyA*Omega*sin(Pdgrd) - 4*AdeA^2*Atrsc*KdeA*Kdgrd^2*KpolyA*cos(2*PdeA) - 4*Adgrd^2*Atrsc*KdeA*Kdgrd^2*KpolyA*cos(2*Pdgrd)
- 4*AdeA^2*Atrsc*KdeA*Kdgrd*Omega^2*cos(2*PdeA) + 2*AdeA^2*Adgrd^2*Atrsc*KdeA*Kdgrd^3*cos(2*PdeA) - 4*AdeA^2*Adgrd*KdeA*Kdgrd*KpolyA*Omega*sin(Pdgrd) + AdeA^2*Adgrd^2*Atrsc*KdeA*Kdgrd^2*KpolyA*cos(2*PdeA)
+ AdeA^2*Adgrd^2*Atrsc*KdeA*Kdgrd^2*KpolyA*cos(2*Pdgrd) + 4*AdeA^2*Adgrd*KdeA*Kdgrd^2*KpolyA*cos(2*PdeA)*cos(Pdgrd) - 4*AdeA^2*ApolyA*KdeA*Kdgrd^2*KpolyA*cos(2*PdeA)*cos(PpolyA) -
4*AdeA^2*ApolyA*KdeA*KpolyA*Omega^2*cos(2*PdeA)*cos(PpolyA) + 4*AdeA*Adgrd^2*Kdgrd^2*KpolyA*Omega*cos(2*Pdgrd)*sin(PdeA) - 4*AdeA*Adgrd^2*Kdgrd^2*KpolyA*Omega*sin(2*Pdgrd)*cos(PdeA) -
4*AdeA*ApolyA^2*Kdgrd*KpolyA^2*Omega*cos(2*PpolyA)*sin(PdeA) + 4*AdeA*ApolyA^2*Kdgrd*KpolyA^2*Omega*sin(2*PpolyA)*cos(PdeA) + 4*AdeA^2*Adgrd*KdeA*Kdgrd^2*KpolyA*sin(2*PdeA)*sin(Pdgrd) -
4*AdeA^2*ApolyA*KdeA*Kdgrd^2*KpolyA*sin(2*PdeA)*sin(PpolyA) - 4*AdeA^2*ApolyA*KdeA*KpolyA*Omega^2*sin(2*PdeA)*sin(PpolyA) + 4*AdeA^2*Atrsc*KdeA*Kdgrd*KpolyA*Omega*sin(2*PdeA) +
4*AdeA^2*Adgrd*KdeA*Kdgrd*KpolyA*Omega*cos(2*PdeA)*sin(Pdgrd) - 4*AdeA^2*Adgrd*KdeA*Kdgrd*KpolyA*Omega*sin(2*PdeA)*cos(Pdgrd) - AdeA^2*Adgrd^2*Atrsc*KdeA*Kdgrd^2*KpolyA*cos(2*PdeA)*cos(2*Pdgrd) -
AdeA^2*Adgrd^2*Atrsc*KdeA*Kdgrd^2*KpolyA*sin(2*PdeA)*sin(2*Pdgrd) - 8*AdeA*Adgrd*Atrsc*KdeA*Kdgrd^2*KpolyA*cos(PdeA)*cos(Pdgrd) + 8*AdeA*ApolyA*Atrsc*KdeA*Kdgrd^2*KpolyA*cos(PdeA)*cos(PpolyA) +
8*Adgrd*ApolyA*Atrsc*KdeA*Kdgrd^2*KpolyA*cos(Pdgrd)*cos(PpolyA) - 2*AdeA*Adgrd^2*ApolyA*Atrsc*KdeA*Kdgrd^2*KpolyA*cos(PdeA)*cos(PpolyA) -
2*AdeA^2*Adgrd*ApolyA*Atrsc*KdeA*Kdgrd^2*KpolyA*cos(Pdgrd)*cos(PpolyA) - 8*AdeA*Adgrd*ApolyA*KdeA*Kdgrd^2*KpolyA*cos(PdeA)*cos(Pdgrd)*cos(PpolyA) -
8*AdeA*Adgrd*ApolyA*Kdgrd*KpolyA^2*Omega*cos(PdeA)*cos(PpolyA)*sin(Pdgrd) + 8*AdeA*Adgrd*ApolyA*Kdgrd^2*KpolyA*Omega*cos(PdeA)*cos(Pdgrd)*sin(PpolyA) -
16*AdeA*Adgrd*ApolyA*Kdgrd^2*KpolyA*Omega*cos(Pdgrd)*cos(PpolyA)*sin(PdeA) - 8*AdeA*Adgrd*ApolyA*KdeA*Kdgrd^2*KpolyA*cos(PdeA)*sin(Pdgrd)*sin(PpolyA) -
8*AdeA*Adgrd*ApolyA*Kdgrd*KpolyA*Omega^2*cos(PdeA)*sin(Pdgrd)*sin(PpolyA) + 8*AdeA*Adgrd*ApolyA*Kdgrd*KpolyA*Omega^2*cos(Pdgrd)*sin(PdeA)*sin(PpolyA) -
8*AdeA*Adgrd*ApolyA*Kdgrd*KpolyA^2*Omega*sin(PdeA)*sin(Pdgrd)*sin(PpolyA) - 8*AdeA*Adgrd*ApolyA*Kdgrd^2*KpolyA*Omega*sin(PdeA)*sin(Pdgrd)*sin(PpolyA) +
8*AdeA*Adgrd*Atrsc*KdeA*Kdgrd*KpolyA*Omega*cos(PdeA)*sin(Pdgrd) - 8*AdeA*ApolyA*Atrsc*KdeA*Kdgrd*KpolyA*Omega*cos(PdeA)*sin(PpolyA) - 8*Adgrd*ApolyA*Atrsc*KdeA*Kdgrd*KpolyA*Omega*cos(PpolyA)*sin(Pdgrd) +
2*AdeA^2*Adgrd*ApolyA*Atrsc*KdeA*Kdgrd*KpolyA*Omega*cos(PpolyA)*sin(Pdgrd) + 8*AdeA*Adgrd*ApolyA*KdeA*Kdgrd*KpolyA*Omega*cos(PdeA)*cos(Pdgrd)*sin(PpolyA) -
8*AdeA*Adgrd*ApolyA*KdeA*Kdgrd*KpolyA*Omega*cos(PdeA)*cos(PpolyA)*sin(Pdgrd) + 2*AdeA*Adgrd^2*ApolyA*Atrsc*KdeA*Kdgrd^2*KpolyA*cos(2*Pdgrd)*cos(PdeA)*cos(PpolyA) +
2*AdeA^2*Adgrd*ApolyA*Atrsc*KdeA*Kdgrd^2*KpolyA*cos(2*PdeA)*cos(Pdgrd)*cos(PpolyA) + 2*AdeA*Adgrd^2*ApolyA*Atrsc*KdeA*Kdgrd^2*KpolyA*sin(2*Pdgrd)*cos(PdeA)*sin(PpolyA) +
2*AdeA^2*Adgrd*ApolyA*Atrsc*KdeA*Kdgrd^2*KpolyA*sin(2*PdeA)*cos(Pdgrd)*sin(PpolyA) - 2*AdeA^2*Adgrd*ApolyA*Atrsc*KdeA*Kdgrd*KpolyA*Omega*cos(2*PdeA)*cos(PpolyA)*sin(Pdgrd) -
2*AdeA^2*Adgrd*ApolyA*Atrsc*KdeA*Kdgrd*KpolyA*Omega*sin(2*PdeA)*sin(Pdgrd)*sin(PpolyA)
```

and B_1 equals

```
8*Adgrd^2*Atrsc*Kdgrd^3*Omega - 16*Atrsc*Kdgrd^3*Omega - 16*Atrsc*Kdgrd*Omega^3 + 16*AdeA*Kdgrd*Omega^3*cos(PdeA) + 16*AdeA*Kdgrd^3*Omega*cos(PdeA) + 16*AdeA*KpolyA*Omega^3*cos(PdeA) -
16*ApolyA*KpolyA*Omega^3*cos(PpolyA) - 16*AdeA*KdeA*Kdgrd^3*sin(PdeA) - 16*AdeA*KpolyA^2*Omega^2*sin(PdeA) + 16*ApolyA*KpolyA^2*Omega^2*sin(PpolyA) - 16*Atrsc*Kdgrd*KpolyA^2*Omega -
32*Atrsc*Kdgrd^2*KpolyA*Omega + 4*AdeA^2*Atrsc*KdeA*Kdgrd^3*sin(2*PdeA) + 4*AdeA*ApolyA^2*KpolyA^2*Omega^2*sin(PdeA) + 4*AdeA*ApolyA^2*KpolyA^2*Omega^2*sin(PdeA - 2*PpolyA) +
8*Adgrd^2*Atrsc*Kdgrd^2*KpolyA*Omega + 16*AdeA*Kdgrd*KpolyA^2*Omega*cos(PdeA) + 32*AdeA*Kdgrd^2*KpolyA*Omega*cos(PdeA) + 16*Adgrd*Kdgrd*KpolyA^2*Omega*cos(Pdgrd) + 16*Adgrd*Kdgrd^2*KpolyA*Omega*cos(Pdgrd)
- 16*ApolyA*Kdgrd*KpolyA^2*Omega*cos(PpolyA) - 16*ApolyA*Kdgrd^2*KpolyA*Omega*cos(PpolyA) - 16*AdeA*KdeA*Kdgrd^2*KpolyA*sin(PdeA) - 16*Adgrd*KdeA*Kdgrd^2*KpolyA*sin(Pdgrd) +
16*ApolyA*KdeA*Kdgrd^2*KpolyA*sin(PpolyA) - 16*AdeA*KdeA*Kdgrd*Omega^2*sin(PdeA) - 16*AdeA*KdeA*KpolyA*Omega^2*sin(PdeA) - 16*AdeA*Kdgrd*KpolyA*Omega^2*sin(PdeA) + 16*Adgrd*Kdgrd*KpolyA*Omega^2*sin(Pdgrd)
+ 16*ApolyA*KdeA*KpolyA*Omega^2*sin(PpolyA) - 16*Atrsc*KdeA*Kdgrd*KpolyA*Omega - 8*AdeA*Adgrd^2*Kdgrd^3*Omega*cos(PdeA) + 8*AdeA*Adgrd^2*KdeA*Kdgrd^3*sin(PdeA) -
4*AdeA*Adgrd^2*Kdgrd^2*KpolyA*Omega*cos(PdeA) - 4*AdeA*ApolyA^2*Kdgrd*KpolyA^2*Omega*cos(PdeA) + 4*AdeA^2*Adgrd*KdeA*Kdgrd^2*KpolyA*sin(Pdgrd) - 4*AdeA^2*ApolyA*KdeA*Kdgrd^2*KpolyA*sin(PpolyA) -
4*AdeA*Adgrd^2*Kdgrd^2*KpolyA*Omega*cos(PdeA - 2*Pdgrd) + 4*AdeA*ApolyA^2*Kdgrd*KpolyA^2*Omega*cos(PdeA - 2*PpolyA) - 4*AdeA^2*ApolyA*KdeA*KpolyA*Omega^2*sin(PpolyA) +
4*AdeA^2*Atrsc*KdeA*Kdgrd*KpolyA*Omega + 16*Adgrd*KdeA*Kdgrd*KpolyA*Omega*cos(Pdgrd) + 4*AdeA^2*Atrsc*KdeA*Kdgrd^2*KpolyA*sin(2*PdeA) + 4*Adgrd^2*Atrsc*KdeA*Kdgrd^2*KpolyA*sin(2*Pdgrd) +
4*AdeA^2*Atrsc*KdeA*Kdgrd*Omega^2*sin(2*PdeA) - 4*AdeA^2*Adgrd*KdeA*Kdgrd^2*KpolyA*sin(2*PdeA - Pdgrd) + 4*AdeA^2*ApolyA*KdeA*Kdgrd^2*KpolyA*sin(2*PdeA - PpolyA) +
4*AdeA^2*ApolyA*KdeA*KpolyA*Omega^2*sin(2*PdeA - PpolyA) - 2*AdeA^2*Adgrd^2*Atrsc*KdeA*Kdgrd^3*sin(2*PdeA) - 4*AdeA^2*Adgrd*KdeA*Kdgrd*KpolyA*Omega*cos(Pdgrd) -
8*AdeA*Adgrd*Atrsc*Kdgrd*KpolyA^2*Omega*cos(PdeA - Pdgrd) - 8*AdeA*Adgrd*Atrsc*Kdgrd^2*KpolyA*Omega*cos(PdeA - Pdgrd) + 8*AdeA*ApolyA*Atrsc*Kdgrd*KpolyA^2*Omega*cos(PdeA - PpolyA) +
8*AdeA*ApolyA*Atrsc*Kdgrd^2*KpolyA*Omega*cos(PdeA - PpolyA) + 8*Adgrd*ApolyA*Atrsc*Kdgrd*KpolyA^2*Omega*cos(Pdgrd - PpolyA) + 8*Adgrd*ApolyA*Atrsc*Kdgrd^2*KpolyA*Omega*cos(Pdgrd - PpolyA) +
4*AdeA*Adgrd*Atrsc*KdeA*Kdgrd^2*KpolyA*sin(PdeA - Pdgrd) - 4*AdeA*ApolyA*Atrsc*KdeA*Kdgrd^2*KpolyA*sin(PdeA - PpolyA) + 4*Adgrd*ApolyA*Atrsc*KdeA*Kdgrd^2*KpolyA*sin(Pdgrd - PpolyA) -
8*AdeA*Adgrd*Atrsc*Kdgrd*KpolyA*Omega^2*sin(PdeA - Pdgrd) + 8*AdeA*ApolyA*Atrsc*Kdgrd*KpolyA*Omega^2*sin(PdeA - PpolyA) - 8*Adgrd*ApolyA*Atrsc*Kdgrd*KpolyA*Omega^2*sin(Pdgrd - PpolyA) -
AdeA^2*Adgrd^2*Atrsc*KdeA*Kdgrd^2*KpolyA*sin(2*PdeA) - AdeA^2*Adgrd^2*Atrsc*KdeA*Kdgrd^2*KpolyA*sin(2*Pdgrd) + AdeA^2*Adgrd^2*Atrsc*KdeA*Kdgrd^2*KpolyA*sin(2*PdeA - 2*Pdgrd) -
4*AdeA*Adgrd*ApolyA*Kdgrd*KpolyA^2*Omega*cos(PdeA + Pdgrd - PpolyA) - 4*AdeA*Adgrd*ApolyA*Kdgrd*KpolyA^2*Omega*cos(Pdgrd - PdeA + PpolyA) - 8*AdeA*Adgrd*ApolyA*Kdgrd^2*KpolyA*Omega*cos(PdeA + Pdgrd -
PpolyA) - 4*AdeA*Adgrd*ApolyA*Kdgrd^2*KpolyA*Omega*cos(PdeA - Pdgrd + PpolyA) + 4*AdeA*Adgrd*ApolyA*Kdgrd^2*KpolyA*Omega*cos(Pdgrd - PdeA + PpolyA) + 4*AdeA^2*Atrsc*KdeA*Kdgrd*KpolyA*Omega*cos(2*PdeA) +
4*AdeA*Adgrd*ApolyA*KdeA*Kdgrd^2*KpolyA*sin(PdeA + Pdgrd - PpolyA) + 4*AdeA*Adgrd*ApolyA*KdeA*Kdgrd^2*KpolyA*sin(PdeA - Pdgrd + PpolyA) + 4*AdeA*Adgrd*ApolyA*Kdgrd*KpolyA*Omega^2*sin(PdeA - Pdgrd +
PpolyA) - 4*AdeA*Adgrd*ApolyA*Kdgrd*KpolyA*Omega^2*sin(Pdgrd - PdeA + PpolyA) - 4*AdeA^2*Adgrd*KdeA*Kdgrd*KpolyA*Omega*cos(2*PdeA - Pdgrd) + 4*AdeA*Adgrd*Atrsc*KdeA*Kdgrd^2*KpolyA*sin(PdeA + Pdgrd) -
4*AdeA*ApolyA*Atrsc*KdeA*Kdgrd^2*KpolyA*sin(PdeA + PpolyA) - 4*Adgrd*ApolyA*Atrsc*KdeA*Kdgrd^2*KpolyA*sin(Pdgrd + PpolyA) - 4*AdeA*Adgrd*Atrsc*KdeA*Kdgrd*KpolyA*Omega*cos(PdeA - Pdgrd) +
4*AdeA*ApolyA*Atrsc*KdeA*Kdgrd*KpolyA*Omega*cos(PdeA - PpolyA) + 4*Adgrd*ApolyA*Atrsc*KdeA*Kdgrd*KpolyA*Omega*cos(Pdgrd - PpolyA) - 2*AdeA*Adgrd*ApolyA^2*Atrsc*Kdgrd*KpolyA^2*Omega*cos(PdeA + Pdgrd -
2*PpolyA) + 2*AdeA*Adgrd^2*ApolyA*Atrsc*Kdgrd^2*KpolyA*Omega*cos(PdeA - 2*Pdgrd + PpolyA) - AdeA*Adgrd^2*ApolyA*Atrsc*KdeA*Kdgrd^2*KpolyA*sin(PdeA - 2*Pdgrd + PpolyA) +
AdeA^2*Adgrd*ApolyA*Atrsc*KdeA*Kdgrd^2*KpolyA*sin(Pdgrd - 2*PdeA + PpolyA) - 4*AdeA*Adgrd*ApolyA*KdeA*Kdgrd*KpolyA*Omega*cos(PdeA + Pdgrd - PpolyA) + 4*AdeA*Adgrd*ApolyA*KdeA*Kdgrd*KpolyA*Omega*cos(PdeA -
Pdgrd + PpolyA) + AdeA*Adgrd^2*ApolyA*Atrsc*KdeA*Kdgrd^2*KpolyA*sin(PdeA + PpolyA) + AdeA^2*Adgrd*ApolyA*Atrsc*KdeA*Kdgrd^2*KpolyA*sin(Pdgrd + PpolyA) -
AdeA*Adgrd^2*ApolyA*Atrsc*KdeA*Kdgrd^2*KpolyA*sin(PdeA + 2*Pdgrd - PpolyA) - AdeA^2*Adgrd*ApolyA*Atrsc*KdeA*Kdgrd^2*KpolyA*sin(2*PdeA + Pdgrd - PpolyA) +
4*AdeA*Adgrd*Atrsc*KdeA*Kdgrd*KpolyA*Omega*cos(PdeA + Pdgrd) - 4*AdeA*ApolyA*Atrsc*KdeA*Kdgrd*KpolyA*Omega*cos(PdeA + PpolyA) - 4*Adgrd*ApolyA*Atrsc*KdeA*Kdgrd*KpolyA*Omega*cos(Pdgrd + PpolyA) +
2*AdeA*Adgrd*ApolyA^2*Atrsc*Kdgrd*KpolyA^2*Omega*cos(PdeA - Pdgrd) - 2*AdeA*Adgrd^2*ApolyA*Atrsc*Kdgrd^2*KpolyA*Omega*cos(PdeA - PpolyA) + AdeA*Adgrd^2*ApolyA*Atrsc*KdeA*Kdgrd^2*KpolyA*sin(PdeA - PpolyA)
- AdeA^2*Adgrd*ApolyA*Atrsc*KdeA*Kdgrd^2*KpolyA*sin(Pdgrd - PpolyA) + AdeA^2*Adgrd*ApolyA*Atrsc*KdeA*Kdgrd*KpolyA*Omega*cos(Pdgrd - 2*PdeA + PpolyA) +
AdeA^2*Adgrd*ApolyA*Atrsc*KdeA*Kdgrd*KpolyA*Omega*cos(Pdgrd + PpolyA) - AdeA^2*Adgrd*ApolyA*Atrsc*KdeA*Kdgrd*KpolyA*Omega*cos(2*PdeA + Pdgrd - PpolyA) -
AdeA^2*Adgrd*ApolyA*Atrsc*KdeA*Kdgrd*KpolyA*Omega*cos(Pdgrd - PpolyA)
```

Since these analytic solutions did not provide much insight about the behaviors of the system, we resorted to numeric solutions.

We have added the following clarification for the choice of method on pp. 8-9.

"Because the parameters of the model are largely unknown and likely vary significantly from gene to gene, we need to investigate the dependency of output rhythmicities on input rhythmicities in the global parameter space (i.e., the entire possible range of parameter values). In previous studies, such dependency was analyzed by deriving approximate analytic solutions to models with up to two rhythmic input processes (Luck et al., 2014; Thurley et al., 2017). With four rhythmic input processes in our model, the approximate analytic solution obtained using the same method as in (Luck et al., 2014; Thurley et al., 2017) are too complex to deliver any useful insight. We hence chose numeric simulations to investigate the input-output dependency for our model."

6. I appreciate the analysis of random parameters since few quantitative details are available. This approach of studying parameter sets instead of specific parameters became popular in chronobiology and the authors might cite papers using this approach: J. Locke BMC 2008, P. Pett LSA 2018, B. Ananthasubramaniam JMB 2020).

Response: We thank the reviewer for the compliment on our methodology. We have added a discussion about generalizability of the methodology on p. 22, and cited these works as suggested.

"Finally, the methodology used in this study, namely, global parameter sensitivity analysis over randomized model parameters, are broadly applicable to modeling studies in chronobiology. Randomized global parameter sweeping is effective and efficient for model analysis, when the model parameters are largely unknown or highly variant (e.g., high variations across different genes for parameters in our model), and the corresponding experimental data are too sparse to effectively constrain the parameters. Results from global parameter sweep provide insights about which elements of the system are important for the target qualitative or quantitative behaviors. Many chronobiology models fall in this type. In fact, similar randomized global parameter sweep was used to identify components that are critical to generate key characteristics of the circadian clock, such as circadian entrainment, adaptation to seasonal

changes in photoperiod, and tissue specific rhythms (Ananthasubramaniam et al., 2020; Locke et al., 2008; Pett et al., 2018)."

Motivated by the reviewer's comment, we also added a brief statement in the model setup section to clarify the expected high variations of model parameters across different genes. This is on p. 7: "Note that the four processes can assume different amplitudes and phases for different genes, because these regulations can be mediated by different combinations of *cis*-elements and *trans*-factors (Charlesworth et al., 2013; Grigull et al., 2004; Koike et al., 2012; Schoenberg and Maquat, 2012; Sun et al., 2013; Ueda et al., 2005)."

7. Regarding Figure 2 I am surprised that DeA controls L/S and L strongly but S only weakly. Typically, an oscillating denominator (S in case of L/S) has a strong impact. Is there any explanation why L seems more relevant regarding DeA regulation.

Response: We suspect that the reviewer meant L+S instead of S, as Fig. 2 did not show any data for S. To answer the reviewer's question about the effect of deadenylation on S, we calculated the Sobol indices for S (Fig. R3 below), and found that deadenylation also strongly controls the rhythmicity of S, as the reviewer expected. In the manuscript, we only presented the results for L, because poly(A) tail facilitates translation initiation and in this work we use L as a rough proxy for mRNA translatability.

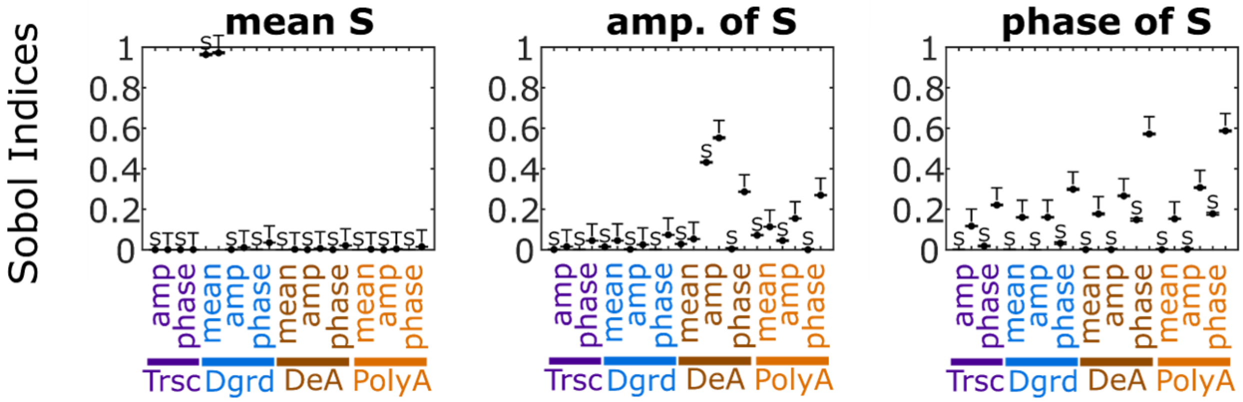

Figure R3: Sobol indices for output S.

8. Looking at Figure 1, I am not surprised that Deadenylation plays a major role. It has a central position and modulates the decay of L and the production of S. If this is the correct interpretation, I would expect for constant DeA a major control of Polyadenylation on the L/S ratio since this reaction is also at the center of the chain and effects L and S.

Response: We agree that polyadenylation lies at the center of the reaction chain, affecting both long-tailed and short-tailed mRNA like deadenylation. We hereby calculated the Sobol indices when deadenylation is non-rhythmic. In this case, the rhythmicity of L/S ratio is indeed mostly controlled by polyadenylation, as the reviewer expected (Fig. R4 below). Our main results in the paper, interestingly, showed that when both polyadenylation and deadenylation are rhythmic,

deadenylation is stronger than polyadenylation in regulating the rhythmicities of L/S ratio (Figs. 2A, 2B, 3(iv), 3(v)).

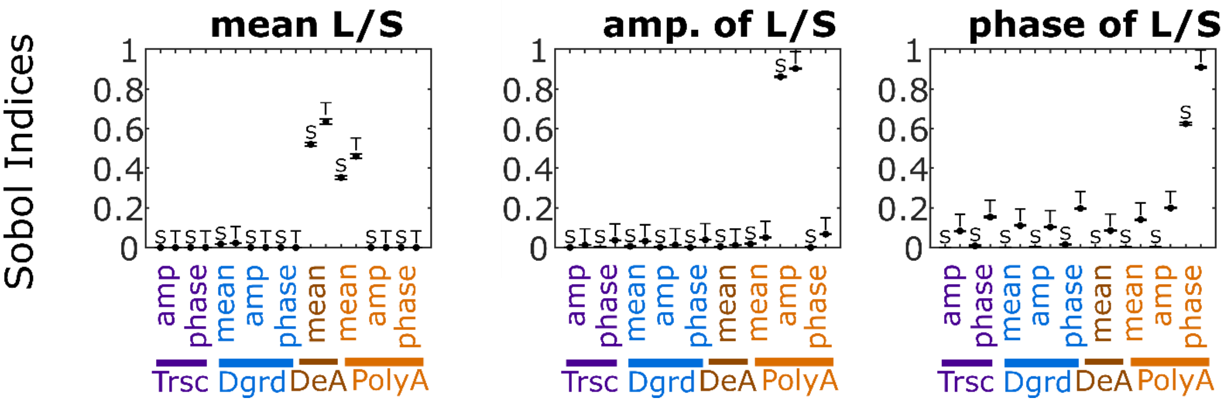

Figure R4: Sobol indices for output L/S for model with constant deadenylation.

List of changes in the manuscript

Main text:

1. In response to Reviewer 3's comment, the Author Summary was significantly revised to achieve a different level of abstraction, and remove redundancy to the Abstract and other parts of the manuscript.
2. The 4 references provided by Reviewer 3 regarding posttranscriptional circadian regulations were added in the 2nd paragraph of Introduction.
3. In response to Reviewer 3's comment, the last paragraph of Introduction was significantly revised to remove redundancy to the other parts of the manuscript.
4. In response to Reviewer 2's comment, the title for the model subsection was revised to: "Model for rhythmic mRNA and poly(A) tail regulation".
5. Motivated by Reviewer 3's comment, a sentence stating the gene-to-gene variations in model parameters was added in the 1st paragraph of the model subsection.
6. In the 1st paragraph of the model subsection, the critical poly(A) tail length that divides the short-tailed and long-tailed mRNAs is corrected from "100 nt" to "60 nt".
7. The last half paragraph of the model subsection was moved to the end of the 1st paragraph of the following subsection to improve the text flow.
8. Following Reviewer 3's suggestion, clarification of the choice of numeric simulation was added to the 1st paragraph of the 2nd subsection of Results.
9. In response to Reviewer 3's comment, the 1st paragraph of Discussion was deleted and partially moved to the end of the current 2nd paragraph (previously 3rd paragraph), in order to reduce text redundancy.
10. The rhythmic fluxes and circadian response analysis suggested by Reviewer 3 were briefly stated in the current 2nd paragraph of the Discussion, and the reference added.
11. Following Reviewer 1's suggestion, the current 5th paragraph of the Discussion was added to discuss the limitation of coarse-graining in the model and potential future work.
12. In response to Reviewer 3's comment, the current 3rd to last paragraph of the Discussion was added to discuss advantage of exploring model behavior using random parameters.
13. Following Reviewer 2's suggestion, the current 2rd to last paragraph of the Discussion was added to discuss advantage of the Sobol's global parameter sensitivity analysis and its potential application to other chronobiology studies.
14. A closing paragraph was added to the end of Discussion.
15. In response to Reviewer 2's comment, more detailed explanation for the Sobol's method was added to the Methods subsection "Sobol's method of global sensitivity analysis".
16. Following Reviewer 2's suggestion, the mistaken subscript in the old Eq. (7) (currently Eq. (8)) was corrected.

Figures:

17. Following Reviewer 1's suggestion, the schematic diagram of the model in Figure 1 was revised to clarify the relation between the core clock machinery and our model.

18. Following Reviewer 1's suggestion, the tick labels in Figure 3 were removed.
19. The red lines in Figs. 4D, 5E were removed, because they did not add any information.

Supplementary Materials:

20. Following Reviewer 2's suggestion, panel labels were added in Figure S1.
21. Figures S4 and S5 were added to clarify the interpretation of Figures 4 and 5 in response to Reviewer 2's comment.
22. Figure S6 was added to compare the Sobol's method with other correlation analyses.

Miscellaneous:

23. All figures, including supplementary figures, have been updated in format (e.g., fonts) to follow the guideline of PLoS Computational Biology.
24. Following Reviewer 3's suggestion, the word "recent" was removed throughout the manuscript when citing Kojima et al., 2012.
25. Following Reviewer 2's suggestion, Figure 1B was referred to on ln. 131, 178, 193, 590 and 594.
26. Miscellaneous text updates were made to correct typos or improve the expression.
